# Evaluation of hospital-acquired conditions reduction program in surgical procedures

**DOI:** 10.1371/journal.pone.0337072

**Published:** 2025-11-21

**Authors:** Yunwei Gai

**Affiliations:** Economics Division, Babson College, Wellesley, Massachusetts, United States of America; Tottori University Faculty of Medicine Graduate School of Medicine: Tottori Daigaku Igakubu Daigakuin Igakukei Kenkyuka, JAPAN

## Abstract

Hospital-Acquired Conditions Reduction Program (HACRP) by the Centers for Medicare & Medicaid (CMS) penalizes hospitals in the worst quartile of performance for targeted hospital-acquired conditions (HAC). This study examines its impacts on two targeted conditions: surgical site infection (SSI) for abdominal hysterectomy and colon procedures. This paper used a quasi-experimental method of difference-in-differences (DID) to analyze the National (Nationwide) Inpatient Sample (NIS) from January 2012 to September 2015. The DID models compared changes in the probability of SSI for the targeted and control procedures before and after HACRP implementation. The DID estimates could be interpreted as the impact of the program. All models controlled for patient and hospital characteristics. The DID estimates were insignificant, and the results were robust across various sensitivity analyses. Although the results suggest that HACRP had no direct impact on SSIs in the two targeted conditions, caution should be exercised when interpreting these conclusions. This study is exploratory, and future research is needed to address its limitations, including the low predictive value of administrative SSI codes and concerns regarding the comparability of the control group. Previous studies have proposed three areas to improve HACRP: better measurement design, improved communication and follow-up, and alternative penalty structure. Collecting more granular data and conducting in-person interviews in future studies could help validate the current findings and identify effective strategies for improving HACRP.

## Background

Surgical site infections (SSIs) are associated with significant increases in morbidity, mortality, and hospital costs. One study found that SSIs at least doubled patients’ mortality risk and cost somewhere between $3.5 billion and $10 billion every year [1]. Because of its severe health and financial consequences, improving surgical care quality is one of the top priorities for various stakeholders in healthcare.

One strategy is public, and sometimes mandatory, reporting of SSI rates. At the state level, some local health authorities have started requiring hospitals to publicly report SSI rates for specific surgical procedures since 2004 [2]. At the national level, Hospital Care Compare, published by the Centers for Medicare & Medicaid (CMS), provides information on hospitals’ quality of care, including SSIs for abdominal hysterectomy and colon procedures.

Another strategy is using financial incentives [3]. The CMS initiated various pay-for-performance (P4P) programs by rewarding or penalizing hospitals based on their quality-of-care performance. Some of the widely publicized programs include the Hospital Value-Based Purchasing Program (HVBPP), Hospital-Acquired Condition Reduction Program (HACRP), and Hospital Readmissions Reduction Program (HRRP).

HACRP, the focus of this study, was launched by CMS in October 2014. Hospitals in the worst-performing quartile for targeted hospital-acquired conditions (HACs), based on Medicare fee-for-service (FFS) claims and chart-abstracted data, receive a 1% reduction in CMS payments for Medicare patients [4]. Since its start, a quarter of hospitals have been penalized, and the total penalties have been over $300 million annually [5]. These penalties could be sizeable for some financially struggling hospitals.

The HACs in the program include two domains. Domain one covers CMS Patient Safety Indicator 90, or PSI 90, based on Medicare claims data. Domain two in HACRP covers central line-associated bloodstream infection (CLABSI), catheter-associated urinary tract infection (CAUTI), SSIs for abdominal hysterectomy and colon procedures, methicillin-resistant Staphylococcus aureus (MRSA) bacteremia, and Clostridium difficile Infection (CDI). Rather than claim or other clinical documentation, domain two is from chart-abstracted surveillance data for all patients from the Centers for Disease Control and Prevention’s National Healthcare Safety Network (NHSN).

Previous studies on HACRP have not reached a consensus regarding the program’s effects. Although some studies [6–9] found reductions in overall HACs, others have yielded mixed results, generally pointing to limited impacts and sometimes counterproductive outcomes from HACRP [4,5,10–16]. This study examines two targeted conditions under HACRP: SSIs for abdominal hysterectomy and colon procedures.

## Methods

### Data

Data in this study came from the NIS from January 2012 to September 2015. NIS, developed for the Healthcare Cost and Utilization Project (HCUP), is the largest publicly available all-payer inpatient database designed to produce national estimates. After taking HCUP Data Use Agreement (DUA) training, researchers can then apply for and purchase the NIS database. The sample period in this study was chosen to maintain consistency with the sampling method and coding system. Before 2012, the NIS was a sample of hospitals. After 2012, NIS was a sample of discharges from all hospitals participating in HCUP [17]. Since October 2015, the ICD-9 codes in NIS have been replaced by ICD-10. This coding change caused problems in trend analyses [18,19]. Therefore, the sampling period in this study ensured consistent sampling and coding methods. Consistent with previous studies [3–5,8,10], the population under study consisted of discharged patients 65 years of age and above with Medicare as their primary insurance. Because HACRP penalizes Medicare payments, it should have a direct impact on this patient group [13]. This study was reviewed and approved as exempt by the Brandeis University Institutional Review Board (IRB).

### Treatment and control outcome variables

The DID analyses compare the treatment groups, i.e., outcomes affected by the policy, with control groups, i.e., outcomes not affected by the policy, to evaluate program and policy effects. The treatment outcomes in this study, i.e., HACRP targeted outcomes, were SSIs in abdominal hysterectomy and colon procedures. The ICD codes for procedures and SSI identification were based on prior literature in the same research area [3,14,20–27]. The first targeted procedure, abdominal hysterectomy, was identified by each discharge’s first, i.e., primary, ICD-9 procedure codes of 68.31, 68.39, 68.41, 68.49, 68.61, and 68.69 [20,21,27]. If diagnosis and procedure codes 567.22, 682.2, 998.31, 998.32, 998.51, and 998.59 were ever present in a patient with abdominal hysterectomy procedures, then they were identified as having SSIs [27–29].

The second targeted condition, colon surgeries included primary procedure codes 17.31–17.36, 17.39, 45.03, 45.26, 45.41, 45.49, 45.52, 45.71–45.76, 45.79, 45.81–45.83, 45.92–45.95, 46.03, 46.04, 46.10, 46.11, 46.13, 46.14, 46.43, 46.52, 46.75, 46.76, and 46.94 [20,21,27]. If one of the following ICD-9 codes was ever reported in colon surgeries, then an SSI event was identified: 567.21, 567.22, 567.29, 567.38, 569.5, 596.61, 596.81, 682.2, 879.9, 998.31, 998.32, 998.51, 998.59, 998.6, 54.0, 54.11, 54.19, 86.04, 86.22, and 86.28 [27–29]. Previous studies provided strong support for using these codes in claims data to identify abdominal hysterectomy, colon procedures, and SSIs in these procedures [20,21,27–29].

The following four control groups and their related SSIs are based on previous studies [14,30]. Similar to the treatment group, the first ICD-9 of each discharge, i.e., primary ICD-9 procedure codes, was used to identify these procedures. Like before, all diagnosis codes in a discharge were used to identify an SSI event.

- Laparoscopic cholecystectomy and laparoscopic appendectomy [30] (procedure: 51.23, 51.24, 47.01. SSI: 567, 567.2, 567.21, 567.22, 567.23, 567.29, 567.3, 567.38, 567.39, 567.8, 567.81, 567.89, 567.9, 682.2)
- Orthopedic Procedures [30] (procedure: 81.01–81.08, 81.23, 81.24, 81.31–81.38, 81.83, 81.85. SSI: 996.67, 998.59)
- Cardiac Implantable Electronic Device [30] (procedure: 00.50, 00.51, 00.52, 00.53, 00.54, 37.80, 37.81, 37.82, 37.83, 37.85, 37.86, 37.87, 37.94, 37.96, 37.98, 37.74, 37.75, 37.76, 37.77, 37.79, 37.89. SSI: 996.61, 998.59)
- SSIs in surgical procedures other than abdominal hysterectomy and colon procedures [22–26,30] (SSI: 998.5, 998.51, 998.59, 996.6–996.69)

### DID model and statistical analysis

This study adopted the difference-in-difference (DID) method, a quasi-experimental design to compare pre- versus post-HACRP changes in SSI rates between targeted and control procedures. Multivariable linear probability models were employed as the empirical method to derive the DID estimator, as they allow coefficient estimates to be directly interpreted as changes in probabilities or rates and are considered reliable for assessing average effects [2,31,32].

The outcome, i.e., dependent variable, is a binary variable that equals one if an SSI event occurred to the patient, zero otherwise. The independent variables include an indicator, *Post*, for program period (one if the discharge date was after October 2014, zero before October 2014), and an indicator, *Treat*, for the treatment group (one if the procedure of the patient was targeted by the HACRP program; zero for the control groups). Their interaction, *Treat×Post*, is the DID estimator, i.e., the estimated impact of HACRP.

Additional control variables include patient and hospital characteristics. Patient characteristics include age, gender, race and ethnicity (non-Hispanic white, non-Hispanic black, Hispanic, and other), Elixhauser mortality score, and quartile classification of residents’ estimated median household income in the patient’s ZIP Code. Hospital characteristics include teaching status, ownership (government, private for-profit, or private nonprofit), location (urban or rural), and U.S. Census region (Northeast, Midwest, South, or West). Dummy variables for the month and year of patient discharge are also included in the model to control for trend effects.

Similar methods were used in previous studies evaluating HAC reduction programs [3,9,14,30,33,34]. In a 2019 study, the authors examined HACRP in the State of Michigan, but the authors used different measures of HAC and different treatment and control outcome measures [14]. In a 2024 study [9], the authors employed a DID design with linear regression models to examine the impact of HACRP using hospital-level data. Three previous studies used DID models to analyze a different CMS program for reducing HAC, Hospital-Acquired Conditions Present on Admission (HAC-POA) program. HAC-POA was implemented in 2008, and its targeted conditions were different from HACRP in this study [30,33,34].

### Sensitivity analysis

Various sensitivity analyses were conducted. First, the identification of treatment and control procedures was expanded. Instead of the first, i.e., primary ICD-9 procedure codes, if the relevant ICD-9 procedure codes were EVER present in a patient’s discharge, they were recognized as in the control or treatment group. Second, the first ICD-9 diagnosis code was excluded from identifying SSIs, as it may indicate preexisting infections rather than those from current procedures [22,30]. Ideally, the Present on Admission (POA) modifier should be used. However, since NIS lacks the POA modifier, prior studies have used this exclusion as a proxy for POA in analyzing CMS program impacts on SSIs [22,30].

Third, alternative ICD-9 codes based on previous studies were used to identify SSIs among treatment and control surgical procedures [22,25,26,30,33,34]. The first alternative ICD-9 code for SSIs included 998.5, 998.51, 998.59, 996.69, 567.2–567.29, 567.9, 567.3–567.39, 682.2, and 682.9 [25,26,30]. The second alternative included 998.5, 998.51, 998.59, 996.6–996.69 [22,25,26]. The third alternative included 567.2–567.29, 567.9, 567.3–567.39, 996.69, 998.5–998.59 [22,25,26,30].

Fifth, the DID models were re-estimated by including two more years of NIS data in 2016 and 2017. General Equivalence Mapping (GEM) from the Centers for Medicare & Medicaid Services (CMS) was used to find the matched ICD-9 and ICD-10 codes. To account for the change in coding, I followed a popular methodological adjustment for including data after the ICD transition years [18,35,36]. Segmented linear binomial regression models, i.e., including dummy variables and trends, were used to capture the “immediate intercept change or so-called ‘jump’ with ICD transition, as well as a time trend or slope that is allowed to vary before and after the transition. [35]” The matched ICD-9 and ICD-10 codes are in the supporting information (S1 Table).

Sixth, interrupted time series (ITS) models were used to analyze the two targeted procedures [16]. For each procedure, the ITS model included a trend variable, a dummy variable equal to zero before HACRP and one after HACRP, and an interaction term between the trend and dummy variable. The coefficient on the dummy variable measures the immediate change in the intercept, while the interaction term measures the slope change following the intervention. All models controlled for patient and hospital characteristics. S7 and S8 Tables report coefficient estimates from these models.

Finally, alternative cut-off times for the post-treatment period were used in DID models. The first cut-off time, also used in the main result, was October 2014, when the financial penalties began. The second was January 2013 because, for the HAC calculations, the CDC used infection data starting from January 2013. The third was in August of 2013 when the program was announced. Presumably, hospitals began paying more attention to the SSIs targeted by the program [8,14].

## Results

Table 1 lists the summary statistics for all observations and patient populations by procedures based on their primary (i.e., first) diagnoses. For categorical variables, the number of observations and proportions are reported. For patient age and Elixhauser mortality score, the mean and standard deviation are reported.

**Table 1:** Summary Statistics for All Observations and Patient Populations by Procedures.

	All	Abdominal hysterectomy	Colon surgeries	Lap chole and lap appy^a^	Orthopedic Procedures	CIED^b^	Other Procedures
Characteristic	(n = 8382176)	(n = 12280)	(n = 80405)	(n = 64206)	(n = 89847)	(n = 70384)	(n = 2144146)
Age, mean (SD), y	77.7 (7.8)	72.5 (6.2)	75.6 (7.2)	75.6 (7.3)	72.5 (5.6)	78.5 (7.4)	75.4 (7.2)
Sex							
Female	4735929 (56.5)	12280 (100)	33609 (41.8)	30112 (46.9)	27801 (43.3)	35955 (56.0)	29534 (46.0)
Male	3646246 (43.5)	0.0 (0.0)	46795 (58.2)	34093 (53.1)	36404 (56.7)	28250 (44.0)	34671 (54.0)
Race							
White	6655447 (79.4)	9480 (77.2)	66655 (82.9)	51300 (79.9)	55666 (86.7)	51814 (80.7)	53098 (82.7)
Black	821453 (9.8)	1363 (11.1)	6512 (8.1)	3980 (6.2)	3788 (5.9)	5650 (8.8)	4622 (7.2)
Hispanic	528077 (6.3)	785 (6.4)	4100 (5.1)	5650 (8.8)	2504 (3.9)	3788 (5.9)	3659 (5.7)
Other race	377197 (4.5)	650 (5.3)	3055 (3.8)	3338 (5.2)	2311 (3.6)	2953 (4.6)	2825 (4.4)
Elixhauser mortality score, mean (SD)	7.5 (9.9)	1.6 (7.2)	8.7 (11.2)	5.4 (8.9)	1.4 (6.8)	4.7 (8.0)	5 (9.3)
Hospital size							
Small	1466880 (17.5)	1215 (9.9)	12382 (15.4)	10658 (16.6)	10593 (16.5)	7255 (11.3)	9695 (15.1)
Medium	2355391 (28.1)	3155 (25.7)	23076 (28.7)	19454 (30.3)	16886 (26.3)	16693 (26.0)	17207 (26.8)
Large	4559903 (54.4)	7896 (64.3)	44946 (55.9)	34093 (53.1)	36661 (57.1)	40257 (62.7)	37303 (58.1)
Location/teaching status of hospital							
Rural	1072918 (12.8)	589 (4.8)	9648 (12.0)	7961 (12.4)	3081 (4.8)	3467 (5.4)	5200 (8.1)
Urban teaching	4308438 (51.4)	9197 (74.9)	42534 (52.9)	29598 (46.1)	39679 (61.8)	39871 (62.1)	37560 (58.5)
Urban nonteach	3000819 (35.8)	2492 (20.3)	28222 (35.1)	26645 (41.5)	21444 (33.4)	20866 (32.5)	21444 (33.4)
Hospital census region							
Northeast	1676435 (20)	3082 (25.1)	15598 (19.4)	10850 (16.9)	9117 (14.2)	14574 (22.7)	11813 (18.4)
Midwest	2003340 (23.9)	3070 (25.0)	20101 (25)	14446 (22.5)	15345 (23.9)	15152 (23.6)	15409 (24.0)
South	3243902 (38.7)	4469 (36.4)	32001 (39.8)	27351 (42.6)	28571 (44.5)	23499 (36.6)	24590 (38.3)
West	1458498 (17.4)	1657 (13.5)	12703 (15.8)	11621 (18.1)	11171 (17.4)	10915 (17)	12391 (19.3)
Ownership of hospital							
Public	846599 (10.1)	1289 (10.5)	7397 (9.2)	5714 (8.9)	5264 (8.2)	4751 (7.4)	5778 (9.0)
Nonprofit	6253103 (74.6)	9664 (78.7)	62233 (77.4)	47319 (73.7)	47127 (73.4)	49438 (77)	48989 (76.3)
For-profit	1282472 (15.3)	1326 (10.8)	10774 (13.4)	11171 (17.4)	11813 (18.4)	9951 (15.5)	9438 (14.7)
Median household income quartile by patient zip codes							
Quartile 1	2290888 (27.8)	2985 (24.7)	20445 (25.8)	16745 (26.5)	21330 (24.1)	17852 (25.8)	527265 (25)
Quartile 2	2198547 (26.7)	3036 (25.1)	21709 (27.4)	17205 (27.3)	24309 (27.5)	18253 (26.4)	563924 (26.8)
Quartile 3	1988122 (24.1)	3019 (25.0)	19861 (25.1)	15634 (24.8)	23116 (26.2)	17318 (25.0)	532575 (25.3)
Quartile 4	1759135 (21.4)	3033 (25.1)	17083 (21.6)	13541 (21.5)	19568 (22.2)	15793 (22.8)	484195 (23.0)
Admission year and quarter							
Year 2012 Q1	601548 (7.2)	989 (8.1)	5828 (7.2)	4365 (6.8)	5541 (6.2)	4449 (6.3)	149579 (7.0)
Year 2012 Q2	563766 (6.7)	947 (7.7)	5556 (6.9)	4414 (6.9)	5445 (6.1)	4145 (5.9)	143851 (6.7)
Year 2012 Q3	548916 (6.6)	940 (7.7)	5549 (6.9)	4622 (7.2)	5493 (6.1)	3990 (5.7)	140964 (6.6)
Year 2012 Q4	567750 (6.8)	931 (7.6)	5540 (6.9)	4250 (6.6)	5577 (6.2)	3776 (5.4)	140626 (6.6)
Year 2013 Q1	594144 (7.1)	809 (6.6)	5360 (6.7)	4023 (6.3)	5617 (6.3)	4909 (7.0)	145716 (6.8)
Year 2013 Q2	557849 (6.7)	844 (6.9)	5478 (6.8)	4361 (6.8)	5735 (6.4)	5255 (7.5)	145202 (6.8)
Year 2013 Q3	540416 (6.4)	850 (6.9)	5564 (6.9)	4424 (6.9)	5903 (6.6)	5115 (7.3)	144106 (6.7)
Year 2013 Q4	543230 (6.5)	794 (6.5)	5288 (6.6)	4070 (6.3)	5803 (6.5)	4535 (6.4)	140439 (6.6)
Year 2014 Q1	557019 (6.6)	711 (5.8)	5281 (6.6)	4067 (6.3)	5956 (6.6)	4706 (6.7)	143541 (6.7)
Year 2014 Q2	549917 (6.6)	747 (6.1)	5224 (6.5)	4129 (6.4)	6132 (6.8)	5022 (7.1)	142724 (6.7)
Year 2014 Q3	534802 (6.4)	732 (6.0)	5178 (6.4)	4226 (6.6)	6340 (7.1)	4850 (6.9)	142144 (6.6)
Year 2014 Q4	555076 (6.6)	705 (5.7)	4959 (6.2)	3989 (6.2)	6028 (6.7)	4163 (5.9)	138815 (6.5)
Year 2015 Q1	590968 (7.1)	789 (6.4)	5345 (6.6)	4391 (6.8)	6819 (7.6)	5207 (7.4)	145421 (6.8)
Year 2015 Q2	562053 (6.7)	776 (6.3)	5418 (6.7)	4498 (7.0)	6776 (7.5)	5304 (7.5)	145238 (6.8)
Year 2015 Q3	512479 (6.1)	714 (5.8)	4831 (6)	4370 (6.8)	6674 (7.4)	4945 (7.0)	135402 (6.3)

^a^Laparoscopic cholecystectomy and laparoscopic appendectomy ^b^Cardiac Implantable Electronic Device.

Since this study focuses on Medicare patients, the average age is over 70 years. The majority of patients are non-Hispanic whites admitted to medium or large nonprofit hospitals in urban areas. Admission years and quarters are about evenly distributed. There are similar patterns for summary statistics by procedures.

**Fig 1:**
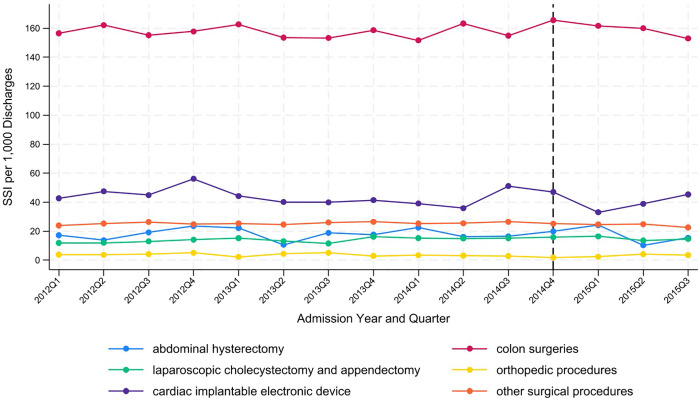
SSI per 1,000 discharges by procedures, admission year and quarter.

Fig 1. presents the number of SSI cases per 1,000 discharges by procedures and admission year and quarter. The corresponding numbers and the percentage of total discharges that underwent the corresponding procedures are provided in the supporting information, S2 Table. The SSI rates for the targeted procedures and control procedures exhibit fluctuations rather than consistent increases or decreases. S3 Table lists the procedure rates and SSI rates when alternative procedures and SSI definitions are used.

Table 2 lists the DID estimates. Reported coefficients are changes per 1,000 discharges. Standard errors, confidence intervals, and the number of observations in each regression are also reported. The cut-off time in the DID models is October 2014, when the financial penalties began. The table only reported the DID estimates, i.e., the interaction term between the post-policy dummy variable and the dummy variable for the treatment procedure. Full regression results are available upon request. All models control for patient and hospital characteristics and time trends. Sample weights and robust standard errors are estimated to generate nationally representative results.

**Table 2:** DID estimates by comparing treatment procedures with control procedures.

	(1)	(2)	(3)	(4)
Abdominal hysterectomy vs.	lap chole and lap appy^a^	orthopedic procedure	CIED^b^	other procedures
Post*treatment	−1.322^c^	0.698	3.132	1.856
	(3.018)^d^	(2.821)	(3.473)	(3.031)
	[-7.237, 4.593]^e^	[-4.831, 6.227]	[-3.675, 9.939]	[-4.085, 7.797]
	71358^f^	94797	76797	1988204
**Colon surgeries vs.**	**lap chole and lap appy** ^**a**^	**orthopedic procedure**	**CIED** ^**b**^	**other procedures**
Post*treatment	3.323	4.247	−2.884	3.986
	(3.300)	(3.160)	(2.607)	(3.194)
	[-3.145, 9.791]	[-1.947, 10.441]	[-7.994, 2.226]	[-2.274, 10.246]
	134980	158419	140419	1988338

^a^Laparoscopic cholecystectomy and laparoscopic appendectomy.

^b^Cardiac Implantable Electronic Device.

^c^coefficient estimate ^d^standard error ^e^95% confidence intervals ^f^number of observations.

For definitions on procedures and SSIs, please refer to the section: Treatment and Control Outcome Variables. All models control for patient and hospital characteristics and time trend.

The top half of the table compares abdominal hysterectomy with each of the four control procedures (cardiac implantable electronic device, laparoscopic cholecystectomy and appendectomy, orthopedic procedures, and all other procedures); the bottom half compares colon surgeries with the four control procedures. The procedures in each comparison are defined by the primary (i.e., the first) ICD-9 procedure codes in each discharge.

Estimates in Table 2 are all insignificant. The DID estimates are largely insignificant in S4 Table when the alternative procedure and SSI identifications are used. The other two cut-off times, January 2013 and August 2013, also have similar results.

Results from additional sensitivity analysis are provided in the supporting information. S1 Fig. presents the number of SSIs per 1,000 discharges, stratified by procedure and admission year/quarter, during the sample period 2012–2017. S5 and S6 Tables report the DID estimates after adding two years of data following the ICD-9 to ICD-10 transition, and the DID estimates remain insignificant. In the results from ITS models (S7 and S8 Tables), neither the HACRP indicator nor its interaction with the trend variable is statistically significant.

## Discussions and conclusions

Prior research on HACRP has yielded mixed evidence regarding the effects of this or similar programs [4–16]. These studies focus on two areas of HACRP: (1) the design and methodology of the program and (2) the impact evaluation of the program. In the first area, several studies found a disconnection between the penalty levied by CMS’ HACRP and the hospital’s quality of care [5,11–13]. Penalties were more likely to be imposed on large teaching hospitals that treated a large proportion of low-income patients [5,10,12]. For example, one study found that the penalty imposed by CMS was primarily driven by the recalibration of PSIs rather than changes in hospitals’ HAC performance [5]. This disconnection may lower hospitals’ incentives and ability to direct resources to reduce SSIs and other HACs. It was found that the risk adjustment method in the HACRP program was inferior to other models [11]. It thus led to the incorrect identification of outlier hospitals and unfairly punished hospitals not based on actual SSI performance [11].

In the second area, i.e., impact evaluation, one study used regression discontinuity design to examine changes in domain one of the HACRP from July 2014 to November 2016 [10]. Another study used pre-program data (January 2009 to September 2014) to simulate potential savings from lowering domain one’s eight HACs [4]. Then, the authors compared these savings with CMS’ savings from payment reduction. Both studies showed no significant relationship between HACRP and improvement in these HACs.

In a 2019 study, the authors used difference-in-differences (DID) and interrupted time series approaches to examine changes in HACs in Michigan hospitals before and after the announcement of HACRP in August 2013 [14]. Due to data limitations, the HACs under study included a subset of domains one and two. The authors did not find any significant associations between patient safety and the HACRP, and the findings questioned the reliability of measures used in the program. A recent study [9] using the DID method and hospital-level data for 2896 hospitals from 2013 to 2020 found significant reductions in HACs following the passage of HACRP. This study has some limitations, such as the relatively short pre-program period available to assess the parallel trend assumption and potential concerns regarding the accuracy of certain HAC scores.

This study examines two targeted conditions: SSIs for abdominal hysterectomy and colon procedures. Consistent with previous studies [4,5,11,14], this paper finds HACRP ineffective in reducing SSIs in the targeted procedures. The trend graph of SSIs in Fig. 1 and the corresponding infection rates in S2 Table show no evidence of a decreasing trend. For example, SSI rates for abdominal hysterectomy procedures fluctuated between 10.31 and 24.08 per 10,000 discharges. Fluctuations around specific mean values are observed for other procedures as well. S3 Table, which reports SSI rates under alternative definitions, likewise shows no clear downward trend. Although these visual observations are informative, formal statistical tests that control for confounding variables are needed for a more conclusive interpretation. The DID estimates in Table 2 are all insignificant, suggesting that HACRP is not significantly associated with changes in SSI rates.

Similar findings are observed in the sensitivity analyses reported in S4 Table, which use alternative definitions for procedures and SSIs. The DID estimates in S4 Table are largely insignificant, except for colon surgeries when SSIs are defined by the third definition: 998.5, 998.51, 998.59, 996.6–996.69. In this case, the DID estimates range from −5.447 to −7.670 depending on the control procedures in the model. These values suggest that HACRP is associated with a reduction of 5.447 to 7.670 SSIs per 1,000 discharges. However, this conclusion may be questioned because the third definition of SSI is more general and less procedure-specific than the other definitions. It omits several ICD codes (e.g., 567.21, 567.22, 567.29, 567.38, 569.5, 596.61, 596.81, 682.2, 879.9) that have been used in prior studies to identify SSI events [27–29].

As a result, SSI rates under the third definition range from 30.22 to 46.33 cases per 1,000 discharges, substantially lower than the 109.29 to 165.56 cases per 1,000 discharges observed under the other two definitions (see S3 Table). This underestimation of SSI rates may in part explain the negative and statistically significant DID estimates found in this sensitivity analysis.

Various sensitivity analyses, including alternative procedure and SSI identification methods, the addition of two years of data after the ICD-9 to ICD-10 transition, and alternative ITS model specifications, yield similar conclusions. Overall, these results suggest no direct effects from HACRP on SSIs in abdominal hysterectomy and colon surgeries.

An important assumption in the DID design is the parallel trend assumption, which requires the treatment and control groups to have similar trends before the intervention. If the parallel trend assumption holds, we have some confidence that both groups would continue to have similar trends had the intervention not occurred. Hence, any deviation from the expected trend can be taken as an indication of the impact of the intervention. The pre-policy trend between treatment and control procedures was analyzed by estimating models with interaction terms between the treatment indicator and discharge year-quarter indicators. The interaction terms are insignificant, suggesting that the trend between the two groups is not significantly different before the policy. Hence, the parallel trend assumption is valid.

This paper makes several contributions to the current literature. First, rather than using associations and trend analyses in some studies, the quasi-experimental DID design was adopted to provide causality analyses. Second, only one previous study used this design to analyze the SSIs in Michigan [14]. This study, by using the National (Nationwide) Inpatient Sample (NIS), provides nationally representative results. Third, to examine the robustness of the results, several definitions of SSIs, control groups, and pre-program periods were used in the analyses.

There are some limitations in this study. One limitation is the accuracy of the measurement by using patient discharge data and ICD-9 codes [4,37]. Unlike some studies that used CPT codes and chart-abstracted surveillance data reported to the NHSN [11], this study used claim-based data and its ICD-9 diagnosis and procedure codes. These codes may not accurately capture the SSI rates because of three problems. The first problem is their low positive predictive value. For instance, a prior study reported a low concordance between medical claims and NHSN surveillance data [37]. If ICD codes underreport (overreport) SSIs for the targeted procedures and overreport (underreport) for the control procedures, the DID estimates would overestimate (underestimate) the impact of HACRP. The second problem is the transition of ICD-9 codes to ICD-10 codes in the fourth quarter of 2015. Despite the use of methodological adjustment, this transition could still affect trend analysis and related DID estimates [18]. The third problem of using ICD codes is the comparability of control groups. Some control procedures may not be clinically appropriate. Moreover, if targeted and control procedures are performed by the same services and improve at similar rates after the passage of HACRP, the DID estimates would fail to capture these improvements.

To address these concerns, this study used four control groups instead of one, each selected based on prior literature in the same research area [3,14,20–27]. The DID models were estimated separately for each control group to provide robustness checks against potential bias from an incorrectly identified control group. In addition, a sensitivity analysis using the ITS models was conducted, offering an additional layer of robustness checks on the findings. Despite these methodological adjustments and sensitivity analyses, concerns remain regarding the accuracy of ICD codes for case identification and the appropriateness of selected control procedures.

Second, the NIS is discharge-level data rather than patient-level data, and it only tracks inpatient hospital stays and does not capture outpatient services. This data structure leads to two problems. First, it fails to account for the changing landscape of hospital care. Hospitals are increasingly shifting toward outpatient services such as emergency room visits and observational stays [38–40]. Because NIS excludes outpatient encounters, these shifts and any related SSIs are not reflected in the analysis. Second, each NIS record represents a single discharge, not an individual patient. As a result, repeated admissions by the same patient are treated as independent observations, since no variables exist to link them. This can bias SSI identification. For example, if a patient underwent colon surgery and was discharged. Later, they were readmitted because of SSI in the first admission. The second admission would not be linked to the first. Instead, its primary diagnosis would differ, preventing the SSI from being attributed to the earlier surgery. Future research could address these limitations by using alternative databases, such as the State Ambulatory Surgery and Services Databases (SASD) from HCUP, which is the largest national database for outpatient visits and outcomes. SASD includes patient identifiers, enabling researchers to track outcomes for the same patient over time, and thus to examine the impact of changes in hospital care on SSIs and other HACs at the patient level.

Finally, this study controls a broad set of patient and hospital characteristics consistent with previous research on HACRP and similar programs [4,8,10,14,22,30]. These confounding factors could affect the infection outcomes. However, the NIS data in this study lack several variables used in the CDC/NHSN risk-adjustment models for calculating SSI scores for HACRP [41]: BMI, procedure duration, wound class, and oncology hospital status. Omission of these variables could bias the estimation results.

Previous studies have proposed several policy recommendations to improve HACRP in three areas: enhancing measurement design, strengthening communication and follow-up, and revising penalty structure. Some studies recommended alternative risk-adjustment models to better account for patient and hospital heterogeneities [11,13,15]. Other studies recommended clearer and more timely communication with hospitals to allow them to make appropriate changes [5,12,14]. They also recommended consistent follow-ups and auditing to improve the program and prevent fraud and misuse. Finally, a 2019 study proposed graduated penalties as in HRRP rather than the all-or-nothing penalty structure in the current HACRP [10]. The authors also suggested changing the threshold for the penalty to accommodate patient mix and other differences in hospital characteristics. Although the NIS used in this study is “the largest publicly available all-payer inpatient care database in the United States [42]” and contains a rich set of patient and hospital-level variables, it lacks detailed information on hospital practices that would allow a deeper analysis of program impact. To address this gap, future research could draw on alternative data sources, such as the American Hospital Association Annual Survey, or employ methods such as in-person interviews with healthcare providers.

In conclusion, although the findings suggest that HACRP had no direct impact on SSIs in the two targeted conditions, these results should be interpreted with caution. This study is exploratory, and future research is needed to address its data limitations. Collecting more granular data and conducting in-person interviews in future studies could help validate the current findings and identify effective strategies for improving HACRP.

### Highlights

- A national study on the impacts of the Hospital-Acquired Conditions Reduction Program.
- Quasi-experimental difference-in-differences model for causal relationship analyses.
- No direct impact of the program on abdominal hysterectomy and colon procedures.
- Robust results across various sensitivity tests.
- Future studies with granular data and interviews could validate findings and guide improvements.

## Supporting information

S1 Fig The number of SSIs per 1,000 discharges by procedures and admission year and quarter (sample period 2012–2017). (JPG)

S1 Table ICD-9/10-CM Codes for Defining the Sample. General Equivalence Mapping (GEM) from the Centers for Medicare & Medicaid Services (CMS) was used to crosswalk between the ICD-10 codes that match the ICD-9 codes used in the paper.
(DOCX)

S2 Table Procedure and SSI Rates by Admission Year and Quarter. Procedure rates are the percentage of total discharges that underwent the corresponding procedures. SSI rates are the number of infections per 1,000 discharges for each procedure.
(DOCX)

S3 Table Procedure and SSI Rates by Alternative Procedures and Definitions by Admission Year and Quarter. (DOCX)

S4 Table DID estimates by comparing treatment procedures to control procedures when alternative procedure and SSI identifications are used. Reported coefficients are changes per 1,000 discharges.
(DOCX)

S5 Table DID estimates by comparing treatment procedures with control procedures (sample period 2012–2017). (DOCX)

S6 Table DID estimates by comparing treatment procedures when alternative procedure and SSI identifications are used (sample period 2012–2017). (DOCX)

S7 Table Interrupted time series for abdominal hysterectomy and colon surgeries (sample period 2012–2015). (DOCX)

S8 Table Interrupted time series for abdominal hysterectomy and colon surgeries (sample period 2012–2017). (DOCX)

## References

[1] Anderson DJ, Podgorny K, Berríos-Torres SI, Bratzler DW, Dellinger EP, Greene L, et al. Strategies to prevent surgical site infections in acute care hospitals: 2014 update. Infect Control Hosp Epidemiol. 2014;35 Suppl 2:S66-88. doi: 10.1017/s0899823x00193869 25376070

[2] Gai Y. Does state-mandated reporting work? the case of surgical site infection in CABG patients. Applied Economics. 2019;51(56):5986–98. doi: 10.1080/00036846.2019.1645282

[3] Kim KM, Max W, White JS, Chapman SA, Muench U. Do penalty-based pay-for-performance programs improve surgical care more effectively than other payment strategies? a systematic review. Ann Med Surg (Lond). 2020;60:623–30. doi: 10.1016/j.amsu.2020.11.060 33304576 PMC7711081

[4] Sankaran R, Gulseren B, Nuliyalu U, Dimick JB, Sheetz K, Arntson E, et al. A comparison of estimated cost savings from potential reductions in hospital-acquired conditions to levied penalties under the cms hospital-acquired condition reduction program. Jt Comm J Qual Patient Saf. 2020;46(8):438–47. doi: 10.1016/j.jcjq.2020.05.002 32571716 PMC8547410

[5] Vsevolozhskaya OA, Manz KC, Zephyr PM, Waters TM. Measurement matters: changing penalty calculations under the hospital acquired condition reduction program (HACRP) cost hospitals millions. BMC Health Serv Res. 2021;21(1):131. doi: 10.1186/s12913-021-06108-w 33563278 PMC7874626

[6] Agency for Healthcare Research and Quality. National Scorecard on Rates of Hospital-Acquired Conditions 2010 to 2015: Interim Data From National Efforts To Make Health Care Safer. 2016. https://www.ahrq.gov/hai/pfp/2015-interim.html

[7] Agency for Healthcare Research and Quality. AHRQ National Scorecard on Hospital-Acquired Conditions Updated Baseline Rates and Preliminary Results 2014–2017. 2019. https://www.ahrq.gov/hai/pfp/index.html

[8] Arntson E, Dimick JB, Nuliyalu U, Errickson J, Engler TA, Ryan AM. Changes in hospital-acquired conditions and mortality associated with the hospital-acquired condition reduction program. Ann Surg. 2021;274(4):e301–7. doi: 10.1097/SLA.0000000000003641 34506324

[9] Wood DM, Beauvais B, Sturdivant RX, Kim FS. Evaluating the effect of financial penalty on hospital-acquired infections. Risk Manag Healthc Policy. 2024;17:2181–90. doi: 10.2147/RMHP.S469424 39263552 PMC11389712

[10] Sankaran R, Sukul D, Nuliyalu U, Gulseren B, Engler TA, Arntson E, et al. Changes in hospital safety following penalties in the US Hospital acquired condition reduction program: retrospective cohort study. BMJ. 2019;366:l4109. doi: 10.1136/bmj.l4109 31270062 PMC6607204

[11] Morgan DM, Kamdar N, Regenbogen SE, Krapohl G, Swenson C, Pearlman M, et al. Evaluation of the methods used by medicare’s hospital-acquired condition reduction program to identify outlier hospitals for surgical site infection. J Am Coll Surg. 2018;227(3):346–56. doi: 10.1016/j.jamcollsurg.2018.06.003 29936061

[12] Fuller RL, Goldfield NI, Averill RF, Hughes JS. Is the CMS Hospital-Acquired Condition Reduction Program a Valid Measure of Hospital Performance?. Am J Med Qual. 2017;32(3):254–60. doi: 10.1177/1062860616640883 27037265

[13] Rajaram R, Chung JW, Kinnier CV, Barnard C, Mohanty S, Pavey ES, et al. Hospital characteristics associated with penalties in the centers for medicare & medicaid services hospital-acquired condition reduction program. JAMA. 2015;314(4):375–83. doi: 10.1001/jama.2015.8609 26219055

[14] Sheetz KH, Dimick JB, Englesbe MJ, Ryan AM. Hospital-acquired condition reduction program is not associated with additional patient safety improvement. Health Aff (Millwood). 2019;38(11):1858–65. doi: 10.1377/hlthaff.2018.05504 31682507

[15] Lawton EJ, Sheetz KH, Ryan AM. Improving the hospital-acquired condition reduction program through rulemaking. JAMA Health Forum. American Medical Association. 2020. p. e200416–e200416. 10.1001/jamahealthforum.2020.0416 PMC8785711 35079737

[16] Lee GM, Kleinman K, Soumerai SB, Tse A, Cole D, Fridkin SK, et al. Effect of nonpayment for preventable infections in U.S. hospitals. N Engl J Med. 2012;367(15):1428–37. doi: 10.1056/NEJMsa1202419 23050526

[17] Agency for Healthcare Research and Quality. HCUP Databases. Rockville, MD: Agency for Healthcare Research and Quality. 2022. http://www.hcup-us.ahrq.gov/nisoverview.jsp

[18] Gibson T, Casto A, Young J, Karnell L, Coenen N. Impact of ICD-10-CM/PCS on Research Using Administrative Databases. U.S. Agency for Healthcare Research and Quality. 2016. http://www.hcup-us.ahrq.gov/reports/methods/methods.jsp

[19] Sebastião YV, Metzger GA, Chisolm DJ, Xiang H, Cooper JN. Impact of ICD-9-CM to ICD-10-CM coding transition on trauma hospitalization trends among young adults in 12 states. Injury Epidemiology. 2021;8(1):1–13. 33487175 10.1186/s40621-021-00298-x PMC7830822

[20] National Healthcare Safety Network. Operative Procedure Categories. Centers for Disease Control and Prevention. https://www.cdc.gov/nhsn/pdfs/operativeprocedures.pdf. 2015.

[21] Department of Health and Human Services. Operational guidance for reporting surgical-site infection (SSI) data to CDC’s NHSN for the purpose of fulfilling CMS’s Hospital Inpatient Quality Reporting (IQR) program requirements. 2015. https://www.cdc.gov/nhsn/PDFs/FINAL-ACH-SSI-Guidance.pdf

[22] Qi AC, Peacock K, Luke AA, Barker A, Olsen MA, Joynt Maddox KE. Associations between social risk factors and surgical site infections after colectomy and abdominal hysterectomy. JAMA Netw Open. 2019;2(10):e1912339. doi: 10.1001/jamanetworkopen.2019.12339 31577353 PMC6777422

[23] de Lissovoy G, Fraeman K, Hutchins V, Murphy D, Song D, Vaughn BB. Surgical site infection: incidence and impact on hospital utilization and treatment costs. Am J Infect Control. 2009;37(5):387–97. doi: 10.1016/j.ajic.2008.12.010 19398246

[24] Saeed MJ, Dubberke ER, Fraser VJ, Olsen MA. Procedure-specific surgical site infection incidence varies widely within certain National Healthcare Safety Network surgery groups. Am J Infect Control. 2015;43(6):617–23. doi: 10.1016/j.ajic.2015.02.012 25818024 PMC4573529

[25] Olsen MA, Nickel KB, Wallace AE, Mines D, Fraser VJ, Warren DK. Stratification of surgical site infection by operative factors and comparison of infection rates after hernia repair. Infect Control Hosp Epidemiol. 2015;36(3):329–35. doi: 10.1017/ice.2014.44 25695175 PMC4683022

[26] Olsen MA, Tian F, Wallace AE, Nickel KB, Warren DK, Fraser VJ, et al. Use of quantile regression to determine the impact on total health care costs of surgical site infections following common ambulatory procedures. Ann Surg. 2017;265(2):331–9. doi: 10.1097/SLA.0000000000001590 28059961 PMC5522732

[27] Calderwood MS, Kleinman K, Bruce CB, Shimelman L, Kaganov RE, Platt R, et al. National validation of the centers for medicare & medicaid services strategy for identifying potential surgical-site infections following colon surgery and abdominal hysterectomy. Infect Control Hosp Epidemiol. 2024;45(2):167–73. doi: 10.1017/ice.2023.193 37675504

[28] Calderwood MS, Huang SS, Keller V, Bruce CB, Kazerouni NN, Janssen L. Variable case detection and many unreported cases of surgical-site infection following colon surgery and abdominal hysterectomy in a statewide validation. Infect Control Hosp Epidemiol. 2017;38(9):1091–7. doi: 10.1017/ice.2017.134 28758616 PMC13043238

[29] Letourneau AR, Calderwood MS, Huang SS, Bratzler DW, Ma A, Yokoe DS. Harnessing claims to improve detection of surgical site infections following hysterectomy and colorectal surgery. Infect Control Hosp Epidemiol. 2013;34(12):1321–3. doi: 10.1086/673975 24225620

[30] Kim KM, White JS, Max W, Chapman SA, Muench U. Evaluation of clinical and economic outcomes following implementation of a medicare pay-for-performance program for surgical procedures. JAMA Netw Open. 2021;4(8):e2121115. doi: 10.1001/jamanetworkopen.2021.21115 34406402 PMC8374611

[31] Gai Y, Pachamanova D. Impact of the medicare hospital readmissions reduction program on vulnerable populations. BMC Health Serv Res. 2019;19(1):837. doi: 10.1186/s12913-019-4645-5 31727168 PMC6857270

[32] Chen M, Grabowski DC. Hospital readmissions reduction program: intended and unintended effects. Med Care Res Rev. 2019;76(5):643–60. doi: 10.1177/1077558717744611 29199504

[33] Thirukumaran CP, Glance LG, Temkin-Greener H, Rosenthal MB, Li Y. Impact of Medicare’s Nonpayment Program on Hospital-acquired conditions. Med Care. 2017;55(5):447–55. doi: 10.1097/MLR.0000000000000680 27922910

[34] Thirukumaran CP, Glance LG, Rosenthal MB, Temkin-Greener H, Balkissoon R, Mesfin A, et al. Impact of medicare’s nonpayment program on venous thromboembolism following hip and knee replacements. Health Serv Res. 2018;53(6):4381–402. doi: 10.1111/1475-6773.13013 30022482 PMC6232432

[35] Hirai AH, Owens PL, Reid LD, Vladutiu CJ, Main EK. Trends in Severe Maternal Morbidity in the US Across the Transition to ICD-10-CM/PCS From 2012-2019. JAMA Netw Open. 2022;5(7):e2222966. doi: 10.1001/jamanetworkopen.2022.22966 35900764 PMC9335134

[36] Salemi JL, Tanner JP, Kirby RS, Cragan JD. The impact of the ICD-9-CM to ICD-10-CM transition on the prevalence of birth defects among infant hospitalizations in the United States. Birth Defects Res. 2019;111(18):1365–79. doi: 10.1002/bdr2.1578 31414582 PMC7194333

[37] Rhee C, Wang R, Jentzsch MS, Broadwell C, Hsu H, Jin R, et al. Comparison of hospital surgical site infection rates and rankings using claims versus National Healthcare Safety Network surveillance data. Infect Control Hosp Epidemiol. 2019;40(2):208–10. doi: 10.1017/ice.2018.310 30509332 PMC9018115

[38] Wadhera RK, Joynt Maddox KE, Kazi DS, Shen C, Yeh RW. Hospital revisits within 30 days after discharge for medical conditions targeted by the Hospital Readmissions Reduction Program in the United States: national retrospective analysis. BMJ. 2019;366:l4563. doi: 10.1136/bmj.l4563 31405902 PMC6689820

[39] Wadhera RK, Yeh RW, Joynt Maddox KE. The Hospital Readmissions Reduction Program - Time for a Reboot. N Engl J Med. 2019;380(24):2289–91. doi: 10.1056/NEJMp1901225 31091367 PMC6589834

[40] Figueroa JF, Wadhera RK. A Decade of observing the hospital readmission reductions program-time to retire an ineffective policy. JAMA Netw Open. 2022;5(11):e2242593. doi: 10.1001/jamanetworkopen.2022.42593 36394876

[41] National Healthcare Safety Network. NHSN Guide to the 2022 Baseline Standardized Infection Ratios. 2022. https://www.cdc.gov/nhsn/2022rebaseline/sir-guide.pdf

[42] Healthcare Cost and Utilization Project (HCUP). HCUP NIS Database Documentation. Rockville, MD: Agency for Healthcare Research and Quality. 2025. http://www.hcup-us.ahrq.gov/db/nation/nis/nisdbdocumentation.jsp 21413206

