# Supplementary figures and images for: Evaluation of hospital-acquired conditions reduction program in surgical procedures
Source: PLoS One. 2025 Nov 21;20(11):e0337072. doi: 10.1371/journal.pone.0337072 (PMC12637954; doi:10.1371/journal.pone.0337072)

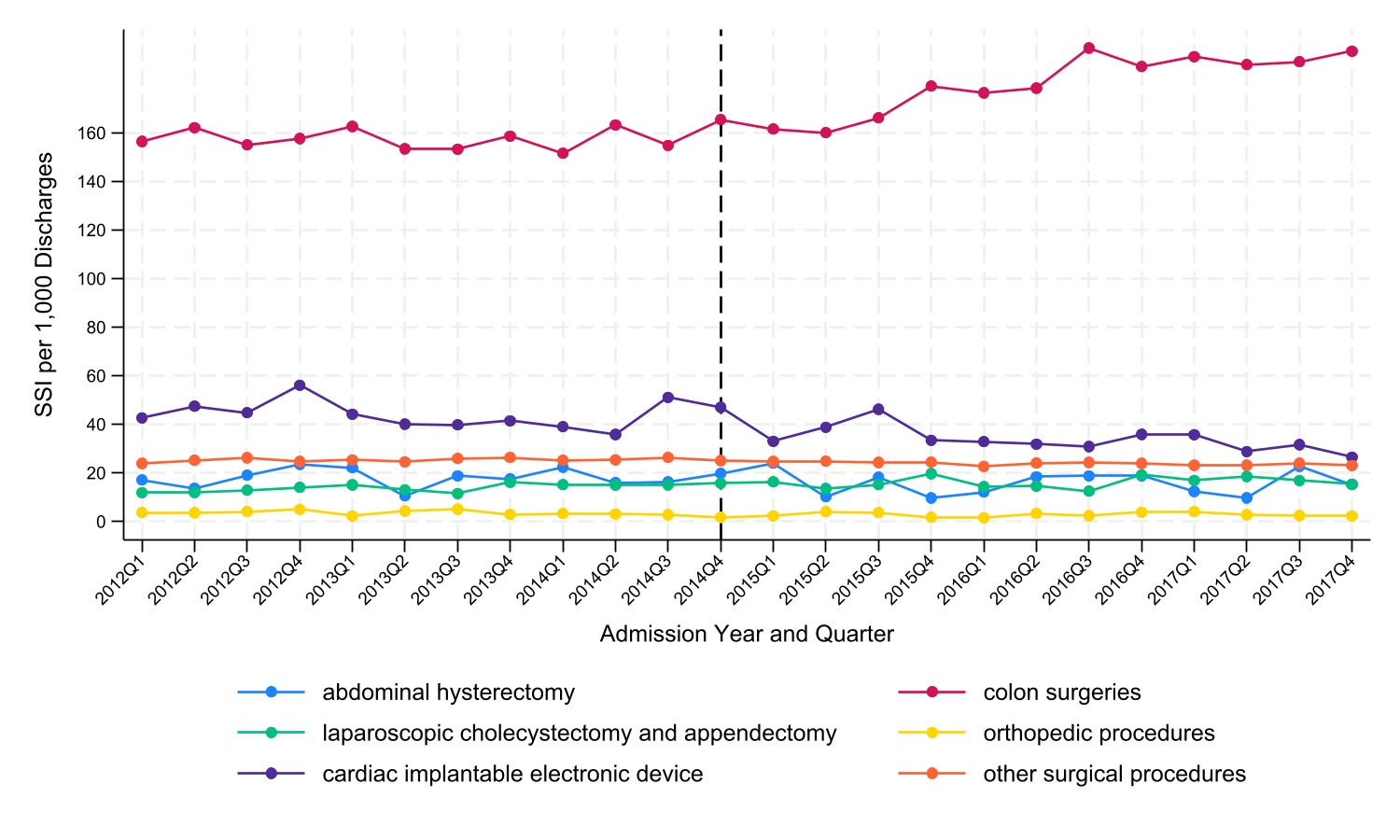

Supplement: S1 Fig — (JPG) [file pone.0337072.s001.jpg]
