# Supplementary material for: Evaluation of hospital-acquired conditions reduction program in surgical procedures
Source: PLoS One. 2025 Nov 21;20(11):e0337072. doi: 10.1371/journal.pone.0337072 (PMC12637954; doi:10.1371/journal.pone.0337072)
Supplement: S1 Table — General Equivalence Mapping (GEM) from the Centers for Medicare & Medicaid Services (CMS) was used to crosswalk between the ICD-10 codes that match the ICD-9 codes used in the paper. (DOCX) [file pone.0337072.s002.docx]

**Supporting information**

S1 Table. is the matched ICD-9 and ICD-10 codes. I used General Equivalence Mapping (GEM) from the Centers for Medicare & Medicaid Services (CMS) to crosswalk between the ICD-10 codes that match the ICD-9 codes used in the paper.

**S1 Table ICD-9/10-CM Codes for Defining the Sample**

| ICD-9/10 Procedure Codes | ICD-9/10 Codes for SSI |
| --- | --- |
| **Abdominal Hysterectomy and related SSI** | |
| 68.31, 68.39, 68.41, 68.49, 68.61, 68.69  0UT94ZZ, 0UT90ZZ, 0UTC4ZZ, 0UTC0ZZ, 0UT44ZZ, 0UT40ZZ | 567.22, 682.2, 998.31, 998.32, 998.51, 998.59  K651, L02219, L03319, L03329, L02211, L03311, L03321, T8132XA, L02212, L03312, L03322, T8131XA, L02213, L03313, L03323, T814XXA, L02214, L03314, L03324, K6811, L02215, L03315, L03325, L03326, L02216, L03316 |
| **Colon Surgeries and related SSI** | |
| 17.31-17.36, 17.39, 45.03, 45.26, 45.41, 45.49, 45.52, 45.71-45.76, 45.79, 45.81-45.83, 45.92-45.95, 46.03, 46.04, 46.10, 46.11, 46.13, 46.14, 46.43, 46.52, 46.75, 46.76, 46.94  0DBE4ZZ, 0D1B47H, 0DBF0ZX, 0D1L87P, 0DCN0ZZ, 0D1H8KM, 0D1B47P  0DTH4ZZ, 0D1B47K, 0DBG0ZX, 0D1L8JL, 0DCN3ZZ, 0D1H8KN, 0D1B4JP  0DTF4ZZ, 0D1B47L, 0DBH0ZX, 0D1L8JM, 0DCN4ZZ, 0D1H8ZH, 0D1B4KP  0DTL4ZZ, 0D1B47M, 0DBK0ZX, 0D1L8JN, 0DPD00Z, 0D1H8ZK, 0D1B4ZP  0DTG4ZZ, 0D1B47N, 0DBL0ZX, 0D1L8JP, 0DPD02Z, 0D1H8ZL, 0D1B87P  0DTN4ZZ, 0D1B4JH, 0DBM0ZX, 0D1L8KL, 0DPD03Z, 0D1H8ZM, 0D1B8JP  0DBF4ZZ, 0D1B4JK, 0DBN0ZX, 0D1L8KM, 0DPD07Z, 0D1H8ZN, 0D1B8KP  0DBG4ZZ, 0D1B4JL, 0DBE0ZZ, 0D1L8KN, 0DPD0CZ, 0D1K07K, 0D1B8ZP  0DBH4ZZ, 0D1B4JM, 0DBE3ZZ, 0D1L8KP, 0DPD0DZ, 0D1K07L, 0D1H87P  0DBK4ZZ, 0D1B4JN, 0DBE7ZZ, 0D1L8ZL, 0DPD0JZ, 0D1K07M, 0D1H8JP  0DBL4ZZ, 0D1B4KH, 0DBF3ZZ, 0D1L8ZM, 0DPD0KZ, 0D1K07N, 0D1H8KP  0DBM4ZZ, 0D1B4KK, 0DBF7ZZ, 0D1L8ZN, 0DPD0UZ, 0D1K07P, 0D1H8ZP  0DBN4ZZ, 0D1B4KL, 0DBG3ZZ, 0D1L8ZP, 0DPD30Z, 0D1K0JK, 0D1907L  0DTK4ZZ, 0D1B4KM, 0DBG7ZZ, 0D1M07M, 0DPD32Z, 0D1K0JL, 0D190JL  0DTM4ZZ, 0D1B4KN, 0DBH0ZZ, 0D1M07N, 0DPD33Z, 0D1K0JM, 0D190KL  0D9E00Z, 0D1B4ZH, 0DBH3ZZ, 0D1M07P, 0DPD37Z, 0D1K0JN, 0D190ZL  0D9E0ZZ, 0D1B4ZK, 0DBH7ZZ, 0D1M0JM, 0DPD3CZ, 0D1K0JP, 0D1947L  0D9E30Z, 0D1B4ZL, 0DBK3ZZ, 0D1M0JN, 0DPD3DZ, 0D1K0KK, 0D194JL  0D9E3ZZ, 0D1B4ZM, 0DBK7ZZ, 0D1M0JP, 0DPD3JZ, 0D1K0KL, 0D194KL  0D9E40Z, 0D1B4ZN, 0DBL3ZZ, 0D1M0KM, 0DPD3KZ, 0D1K0KM, 0D194ZL  0D9E4ZZ, 0D1B87H, 0DBL7ZZ, 0D1M0KN, 0DPD3UZ, 0D1K0KN, 0D1987L  0D9E7ZZ, 0D1B87K, 0DBM3ZZ, 0D1M0KP, 0DPD40Z, 0D1K0KP, 0D198JL  0D9E8ZZ, 0D1B87L, 0DBM7ZZ, 0D1M0ZM, 0DPD42Z, 0D1K0ZK, 0D198KL  0D9F00Z, 0D1B87M, 0DBN3ZZ, 0D1M0ZN, 0DPD43Z, 0D1K0ZL, 0D198ZL  0D9F0ZZ, 0D1B87N, 0DBN7ZZ, 0D1M0ZP, 0DPD47Z, 0D1K0ZM, 0D1A07H  0D9F30Z, 0D1B8JH, 0D5E0ZZ, 0D1M47M, 0DPD4CZ, 0D1K0ZN, 0D1A07K  0D9F3ZZ, 0D1B8JK, 0D5E3ZZ, 0D1M47N, 0DPD4DZ, 0D1K0ZP, 0D1A07L  0D9F40Z, 0D1B8JL, 0D5E7ZZ, 0D1M47P, 0DPD4JZ, 0D1K47K, 0D1A07M  0D9F4ZZ, 0D1B8JM, 0D5F0ZZ, 0D1M4JM, 0DPD4KZ, 0D1K47L, 0D1A07N  0D9F7ZZ, 0D1B8JN, 0D5F3ZZ, 0D1M4JN, 0DPD4UZ, 0D1K47M, 0D1A0JH  0D9F8ZZ, 0D1B8KH, 0D5F7ZZ, 0D1M4JP, 0DPD70Z, 0D1K47N, 0D1A0JK  0D9G00Z, 0D1B8KK, 0D5G0ZZ, 0D1M4KM, 0DPD72Z, 0D1K47P, 0D1A0JL  0D9G0ZZ, 0D1B8KL, 0D5G3ZZ, 0D1M4KN, 0DPD73Z, 0D1K4JK, 0D1A0JM  0D9G30Z, 0D1B8KM, 0D5G7ZZ, 0D1M4KP, 0DPD77Z, 0D1K4JL, 0D1A0JN  0D9G3ZZ, 0D1B8KN, 0D5H0ZZ, 0D1M4ZM, 0DPD7CZ, 0D1K4JM, 0D1A0KH  0D9G40Z, 0D1B8ZK, 0D5H3ZZ, 0D1M4ZN, 0DPD7DZ, 0D1K4JN, 0D1A0KK  0D9G4ZZ, 0D1B8ZL, 0D5H7ZZ, 0D1M4ZP, 0DPD7JZ, 0D1K4JP, 0D1A0KL  0D9G7ZZ, 0D1B8ZM, 0D5K0ZZ, 0D1M87M, 0DPD7KZ, 0D1K4KK, 0D1A0KM  0D9G8ZZ, 0D1B8ZN, 0D5K3ZZ, 0D1M87N, 0DPD7UZ, 0D1K4KL, 0D1A0KN  0D9H00Z, 0D1H07H, 0D5K7ZZ, 0D1M87P, 0DPD80Z, 0D1K4KM, 0D1A0ZH  0D9H0ZZ, 0D1H07K, 0D5L0ZZ, 0D1M8JM, 0DPD82Z, 0D1K4KN, 0D1A0ZK  0D9H30Z, 0D1H07L, 0D5L3ZZ, 0D1M8JN, 0DPD83Z, 0D1K4KP, 0D1A0ZL  0D9H3ZZ, 0D1H07M, 0D5L7ZZ, 0D1M8JP, 0DPD87Z, 0D1K4ZK, 0D1A0ZM  0D9H40Z, 0D1H07N, 0D5M0ZZ, 0D1M8KM, 0DPD8CZ, 0D1K4ZL, 0D1A0ZN  0D9H4ZZ, 0D1H07P, 0D5M3ZZ, 0D1M8KN, 0DPD8DZ, 0D1K4ZM, 0D1A47H  0D9H7ZZ, 0D1H0JH, 0D5M7ZZ, 0D1M8KP, 0DPD8JZ, 0D1K4ZN, 0D1A47K  0D9H8ZZ, 0D1H0JK, 0D5N0ZZ, 0D1M8ZM, 0DPD8KZ, 0D1K4ZP, 0D1A47L  0D9K00Z, 0D1H0JL, 0D5N3ZZ, 0D1M8ZN, 0DPD8UZ, 0D1K87K, 0D1A47M  0D9K0ZZ, 0D1H0JM, 0D5N7ZZ, 0D1M8ZP, 0DWD00Z, 0D1K87L, 0D1A47N  0D9K30Z, 0D1H0JN, 0DBN0ZZ, 0D1N07N, 0DWD02Z, 0D1K87M, 0D1A4JH  0D9K3ZZ, 0D1H0JP, 0TRB07Z, 0D1N07P, 0DWD03Z, 0D1K87N, 0D1A4JK  0D9K40Z, 0D1H0KH, 0DBE8ZZ, 0D1N0JN, 0DWD07Z, 0D1K87P, 0D1A4JL  0D9K4ZZ, 0D1H0KK, 0DTH0ZZ, 0D1N0JP, 0DWD0CZ, 0D1K8JK, 0D1A4JM  0D9K7ZZ, 0D1H0KL, 0DTH7ZZ, 0D1N0KN, 0DWD0DZ, 0D1K8JL, 0D1A4JN  0D9K8ZZ, 0D1H0KM, 0DTH8ZZ, 0D1N0KP, 0DWD0JZ, 0D1K8JM, 0D1A4KH  0D9L00Z, 0D1H0KN, 0DTF0ZZ, 0D1N0ZN, 0DWD0KZ, 0D1K8JN, 0D1A4KK  0D9L0ZZ, 0D1H0KP, 0DTF7ZZ, 0D1N0ZP, 0DWD0UZ, 0D1K8JP, 0D1A4KL  0D9L30Z, 0D1H0ZH, 0DTF8ZZ, 0D1N47N, 0DWD30Z, 0D1K8KK, 0D1A4KM  0D9L3ZZ, 0D1H0ZK, 0DTK0ZZ, 0D1N47P, 0DWD32Z, 0D1K8KL, 0D1A4KN  0D9L40Z, 0D1H0ZL, 0DTL0ZZ, 0D1N4JN, 0DWD33Z, 0D1K8KM, 0D1A4ZH  0D9L4ZZ, 0D1H0ZM, 0DTL7ZZ, 0D1N4JP, 0DWD37Z, 0D1K8KN, 0D1A4ZK  0D9L7ZZ, 0D1H0ZN, 0DTL8ZZ, 0D1N4KN, 0DWD3CZ, 0D1K8KP, 0D1A4ZL  0D9L8ZZ, 0D1H0ZP, 0DTG0ZZ, 0D1N4KP, 0DWD3DZ, 0D1K8ZK, 0D1A4ZM  0D9M00Z, 0D1H47H, 0DTG7ZZ, 0D1N4ZN, 0DWD3JZ, 0D1K8ZL, 0D1A4ZN  0D9M0ZZ, 0D1H47K, 0DTG8ZZ, 0D1N4ZP, 0DWD3KZ, 0D1K8ZM, 0D1A87H  0D9M30Z, 0D1H47L, 0DTN0ZZ, 0D1N87N, 0DWD3UZ, 0D1K8ZN, 0D1A87K  0D9M3ZZ, 0D1H47M, 0DTN7ZZ, 0D1N87P, 0DWD40Z, 0D1K8ZP, 0D1A87L  0D9M40Z, 0D1H47N, 0DTN8ZZ, 0D1N8JN, 0DWD42Z, 0D1L07L, 0D1A87M  0D9M4ZZ, 0D1H47P, 0DBF8ZZ, 0D1N8JP, 0DWD43Z, 0D1L07M, 0D1A87N  0D9M7ZZ, 0D1H4JH, 0DBG8ZZ, 0D1N8KN, 0DWD47Z, 0D1L07N, 0D1A8JH  0D9M8ZZ, 0D1H4JK, 0DBH8ZZ, 0D1N8KP, 0DWD4CZ, 0D1L07P, 0D1A8JK  0D9N00Z, 0D1H4JL, 0DBK8ZZ, 0D1N8ZN, 0DWD4DZ, 0D1L0JL, 0D1A8JL  0D9N0ZZ, 0D1H4JM, 0DBL8ZZ, 0D1N8ZP, 0DWD4JZ, 0D1L0JM, 0D1A8JM  0D9N30Z, 0D1H4JN, 0DBM8ZZ, 0D1A07Q, 0DWD4KZ, 0D1L0JN, 0D1A8JN  0D9N3ZZ, 0D1H4JP, 0DBN8ZZ, 0D1A0JQ, 0DWD4UZ, 0D1L0JP, 0D1A8KH  0D9N40Z, 0D1H4KH, 0DTK7ZZ, 0D1A0KQ, 0DWD70Z, 0D1L0KL, 0D1A8KK  0D9N4ZZ, 0D1H4KK, 0DTK8ZZ, 0D1A0ZQ, 0DWD72Z, 0D1L0KM, 0D1A8KL  0D9N7ZZ, 0D1H4KL, 0DTM0ZZ, 0D1A47Q, 0DWD73Z, 0D1L0KN, 0D1A8KM  0D9N8ZZ, 0D1H4KM, 0DTM7ZZ, 0D1A4JQ, 0DWD77Z, 0D1L0KP, 0D1A8KN  0DCE0ZZ, 0D1H4KN, 0DTM8ZZ, 0D1A4KQ, 0DWD7CZ, 0D1L0ZL, 0D1A8ZK  0DCE3ZZ, 0D1H4KP, 0DTE4ZZ, 0D1A4ZQ, 0DWD7DZ, 0D1L0ZM, 0D1A8ZL  0DCE4ZZ, 0D1H4ZH, 0DTE0ZZ, 0D1A87Q, 0DWD7JZ, 0D1L0ZN, 0D1A8ZM  0DCF0ZZ, 0D1H4ZK, 0DTE7ZZ, 0D1A8JQ, 0DWD7KZ, 0D1L0ZP, 0D1A8ZN  0DCF3ZZ, 0D1H4ZL, 0DTE8ZZ, 0D1A8KQ, 0DWD7UZ, 0D1L47L, 0D1B07H  0DCF4ZZ, 0D1H4ZM, 0D1A07P, 0D1A8ZQ, 0DWD80Z, 0D1L47M, 0D1B07K  0DCG0ZZ, 0D1H4ZN, 0D1A0JP, 0D1B07Q, 0DWD82Z, 0D1L47N, 0D1B07L  0DCG3ZZ, 0D1H4ZP, 0D1A0KP, 0D1B0JQ, 0DWD83Z, 0D1L47P, 0D1B07M  0DCG4ZZ, 0D1H87H, 0D1A0ZP, 0D1B0KQ, 0DWD87Z, 0D1L4JL, 0D1B07N  0DCH0ZZ, 0D1H87K, 0D1A47P, 0D1B0ZQ, 0DWD8CZ, 0D1L4JM, 0D1B0JH  0DCH3ZZ, 0D1H87L, 0D1A4JP, 0D1B47Q, 0DWD8DZ, 0D1L4JN, 0D1B0JK  0DCH4ZZ, 0D1H87M, 0D1A4KP, 0D1B4JQ, 0DWD8JZ, 0D1L4JP, 0D1B0JL  0DCK0ZZ, 0D1H87N, 0D1A4ZP, 0D1B4KQ, 0DWD8KZ, 0D1L4KL, 0D1B0JM  0DCK3ZZ, 0D1H8JH, 0D1A87P, 0D1B4ZQ, 0DWD8UZ, 0D1L4KM, 0D1B0JN  0DCK4ZZ, 0D1H8JK, 0D1A8JP, 0D1B87Q, 0D9E0ZX, 0D1L4KN, 0D1B0KH  0DCL0ZZ, 0D1H8JL, 0D1A8KP, 0D1B8JQ, 0D9F0ZX, 0D1L4KP, 0D1B0KK  0DCL3ZZ, 0D1H8JM, 0D1A8ZP, 0D1B8KQ, 0D9G0ZX, 0D1L4ZL, 0D1B0KL  0DCL4ZZ, 0D1H8JN, 0D1B07P, 0D1B8ZQ, 0D9H0ZX, 0D1L4ZM, 0D1B0KM  0DCM0ZZ, 0D1H8KH, 0D1B0JP, 0D1K0Z4, 0D9K0ZX, 0D1L4ZN, 0D1B0KN  0DCM3ZZ, 0D1H8KK, 0D1B0KP, 0D1K4Z4, 0D9L0ZX, 0D1L4ZP, 0D1B0ZH  0DCM4ZZ, 0D1H8KL, 0D1B0ZP, 0D1K8Z4, 0D9M0ZX, 0D1L87L, 0D1B0ZK  0DBE0ZX, 0D1L87N, 0D1B0ZM, 0DWE8JZ, 0D9N0ZX, 0D1L87M, 0D1B0ZL  0D1B0ZN, 0DWE8KZ, 0WBFXZ2, 0D1K8K4, 0D1N8Z4, 0D1H474, 0D1M4J4  0D1L0Z4, 0D1L0J4, 0WQFXZ2, 0D1L074, 0H87XZZ, 0D1H4J4, 0D1M4K4  0D1L4Z4, 0D1L0K4, 0DQE0ZZ, 0D1H8J4, 0D1M8K4, 0DQN8ZZ, 0DQK7ZZ  0D1L8Z4, 0D1L3J4, 0DQH0ZZ, 0D1H8K4, 0D1M8Z4, 0HQ9XZZ, 0DQK8ZZ  0D1N0Z4, 0D1L474, 0DQN0ZZ, 0D1H8Z4, 0D1N074, 0DWE07Z, 0DQN3ZZ  0D1N4Z4, 0D1L4J4, 0DQE3ZZ, 0D1K074, 0D1N0J4, 0DWE0JZ, 0D1M4Z4  0DBF0ZZ, 0D1L4K4, 0DQE4ZZ, 0D1K0J4, 0D1N0K4, 0DWE0KZ, 0D1H4K4  0DBG0ZZ, 0D1L874, 0DQE7ZZ, 0D1K0K4, 0D1N3J4, 0DWE47Z, 0DWE7JZ  0DBK0ZZ, 0D1L8J4, 0DQE8ZZ, 0D1K3J4, 0D1N474, 0DWE4JZ, 0DWE7KZ  0DBL0ZZ, 0D1L8K4, 0DQH3ZZ, 0D1K474, 0D1N4J4, 0DWE4KZ, 0DWE87Z  0DBM0ZZ, 0D1M074, 0DQH4ZZ, 0D1K4J4, 0D1N4K4, 0DWE77Z, 0DQN4ZZ  0D1H074, 0D1M0J4, 0DQH7ZZ, 0D1K4K4, 0D1N874, 0D1N8J4, 0DQN7ZZ  0D1H0J4, 0D1M0K4, 0DQH8ZZ, 0DQK0ZZ, 0D1K874, 0D1N8K4, 0D1H3J4  0D1H0K4, 0D1M0Z4, 0D1H874, 0DQK3ZZ, 0D1K8J4, 0D1M874, 0D1M8J4  0D1H0Z4, 0D1M3J4, 0D1M474, 0DQK4ZZ, 0D1H4Z4 | 567.21, 567.22, 567.29, 567.38, 569.5, 596.61, 596.81, 682.2, 879.9, 998.31, 998.32, 998.51, 998.59, 998.6, 54.0, 54.11, 54.19, 86.04, 86.22, 86.28  0W9F00Z, 0X924ZZ, 0JBN0ZZ, 0WWF0YZ  0W9F0ZZ, 0X9300Z, 0JBP0ZZ, 0WWF30Z  0W9H00Z, 0X930ZZ, 0JBQ0ZZ, 0WWF31Z  0W9H0ZZ, 0X9330Z, 0JBR0ZZ, 0WWF33Z  0W9H30Z, 0X933ZZ, 0HD0XZZ, 0WWF37Z  0W9H3ZZ, 0X9340Z, 0HD1XZZ, 0WWF3JZ  0W9H40Z, 0X934ZZ, 0HD4XZZ, 0WWF3KZ  0W9H4ZZ, 0X9400Z, 0HD5XZZ, 0WWF3YZ  0WCJ3ZZ, 0X940ZZ, 0HD6XZZ, 0WWF40Z  0WCJ4ZZ, 0X9430Z, 0HD7XZZ, 0WWF41Z  0WCP3ZZ, 0X943ZZ, 0HD8XZZ, 0WWF43Z  0WCP4ZZ, 0X9440Z, 0HD9XZZ, 0WWF47Z  0WCR3ZZ, 0X944ZZ, 0HDAXZZ, 0WWF4JZ  0WCR4ZZ, 0X9500Z, 0HDBXZZ, 0WWF4KZ  0WHF03Z, 0X950ZZ, 0HDCXZZ, 0WWF4YZ  0WHF0YZ, 0X9530Z, 0HDDXZZ, 0Y9500Z  0WHF33Z, 0X953ZZ, 0HDEXZZ, 0Y950ZZ  0WHF3YZ, 0X9540Z, 0HDFXZZ, 0Y9530Z  0WHF43Z, 0X954ZZ, 0HDGXZZ, 0Y953ZZ  0WHF4YZ, 0X9600Z, 0HDHXZZ, 0Y9540Z  0WJF0ZZ, 0X960ZZ, 0HDJXZZ, 0Y954ZZ  0WJH0ZZ, 0X9630Z, 0HDKXZZ, 0Y9600Z  0WPF00Z, 0X963ZZ, 0HDLXZZ, 0Y960ZZ  0WPF01Z, 0X9640Z, 0HDMXZZ, 0Y9630Z  0WPF03Z, 0X964ZZ, 0HDNXZZ, 0Y963ZZ  0WPF07Z, 0X9700Z, 0JD00ZZ, 0Y9640Z  0WPF0JZ, 0X970ZZ, 0JD10ZZ, 0Y964ZZ  0WPF0KZ, 0X9730Z, 0JD40ZZ, 0YJ50ZZ  0WPF0YZ, 0X973ZZ, 0JD50ZZ, 0YJ60ZZ  0WPF30Z, 0X9740Z, 0JD60ZZ, 0YJ70ZZ  0WPF31Z, 0X974ZZ, 0JD70ZZ, 0YJA0ZZ  0WPF33Z, 0X9800Z, 0JD80ZZ, 07JP0ZZ  0WPF37Z, 0X980ZZ, 0JD90ZZ, 0DJ00ZZ  0WPF3JZ, 0X9830Z, 0JDB0ZZ, 0DJ60ZZ  0WPF3KZ, 0X983ZZ, 0JDC0ZZ, 0DJD0ZZ  0WPF3YZ, 0X9840Z, 0JDD0ZZ, 0DJU0ZZ  0WPF40Z, 0X984ZZ, 0JDF0ZZ, 0DJV0ZZ  0WPF41Z, 0X9900Z, 0JDG0ZZ, 0DJW0ZZ  0WPF43Z, 0X990ZZ, 0JDH0ZZ, 0FJ00ZZ  0WPF47Z, 0X9930Z, 0JDJ0ZZ, 0WJG0ZZ  0WPF4JZ, 0X993ZZ, 0JDK0ZZ, 0WJJ0ZZ  0WPF4KZ, 0X9940Z, 0JDL0ZZ, 0WJP0ZZ  0WPF4YZ, 0X994ZZ, 0JDM0ZZ, 0WJR0ZZ  0WWF00Z, 0X9B00Z, 0JDN0ZZ, 0D9S00Z  0WWF01Z, 0X9B0ZZ, 0JDP0ZZ, 0D9S0ZZ  0WWF03Z, 0X9B30Z, 0JDQ0ZZ, 0D9T00Z  0WWF07Z, 0X9B3ZZ, 0JDR0ZZ, 0D9T0ZZ  0WWF0JZ, 0X9B40Z, 3E1038Z, 0D9V00Z  0WWF0KZ, 0X9B4ZZ, 3E10X8Z, 0D9V0ZZ  0Y9L4ZZ, 0X9C00Z, 0Y980ZZ, 0D9W00Z  0Y9M00Z, 0X9C0ZZ, 0Y9830Z, 0D9W0ZZ  0Y9M0ZZ, 0X9C30Z, 0Y983ZZ, 0W9G00Z  0Y9M30Z, 0X9C3ZZ, 0Y9840Z, 0W9G0ZZ  0Y9M3ZZ, 0X9C40Z, 0Y984ZZ, 0WCJ0ZZ  0Y9M40Z, 0X9C4ZZ, 0Y9900Z, 0WCP0ZZ  0Y9M4ZZ, 0X9D00Z, 0Y990ZZ, 0WCR0ZZ  0Y9N00Z, 0X9D0ZZ, 0Y9930Z, 0H90X0Z  0Y9N0ZZ, 0X9D30Z, 0Y993ZZ, 0H90XZZ  0Y9N30Z, 0X9D3ZZ, 0Y9940Z, 0H91X0Z  0Y9N3ZZ, 0X9D40Z, 0Y994ZZ, 0H91XZZ  0Y9N40Z, 0X9D4ZZ, 0Y9B00Z, 0H94X0Z  0Y9N4ZZ, 0X9F00Z, 0Y9B0ZZ, 0H94XZZ  0HB0XZZ, 0X9F0ZZ, 0Y9B30Z, 0H95X0Z  0HB1XZZ, 0X9F30Z, 0Y9B3ZZ, 0H95XZZ  0HB4XZZ, 0X9F3ZZ, 0Y9B40Z, 0H96X0Z  0HB5XZZ, 0X9F40Z, 0Y9B4ZZ, 0H96XZZ  0HB6XZZ, 0X9F4ZZ, 0Y9C00Z, 0H97X0Z  0HB7XZZ, 0X9G00Z, 0Y9C0ZZ, 0H97XZZ  0HB8XZZ, 0X9G0ZZ, 0Y9C30Z, 0H98X0Z  0HBAXZZ, 0X9G30Z, 0Y9C3ZZ, 0H98XZZ  0HBBXZZ, 0X9G3ZZ, 0Y9C40Z, 0H9AX0Z  0HBCXZZ, 0X9G40Z, 0Y9C4ZZ, 0H9AXZZ  0HBDXZZ, 0X9G4ZZ, 0Y9D00Z, 0H9BX0Z  0HBEXZZ, 0X9H00Z, 0Y9D0ZZ, 0H9BXZZ  0HBFXZZ, 0X9H0ZZ, 0Y9D30Z, 0H9CX0Z  0HBGXZZ, 0X9H30Z, 0Y9D3ZZ, 0H9CXZZ  0HBHXZZ, 0X9H3ZZ, 0Y9D40Z, 0H9DX0Z  0HBJXZZ, 0X9H40Z, 0Y9D4ZZ, 0H9DXZZ  0HBKXZZ, 0X9H4ZZ, 0Y9F00Z, 0H9EX0Z  0HBLXZZ, 0Y9000Z, 0Y9F0ZZ, 0H9EXZZ  0HBMXZZ, 0Y900ZZ, 0Y9F30Z, 0H9FX0Z  0HBNXZZ, 0Y9030Z, 0Y9F3ZZ, 0H9FXZZ  0JB00ZZ, 0Y903ZZ, 0Y9F40Z, 0H9GX0Z  0JB10ZZ, 0Y9040Z, 0Y9F4ZZ, 0H9GXZZ  0JB40ZZ, 0Y904ZZ, 0Y9G00Z, 0H9HX0Z  0JB50ZZ, 0Y9100Z, 0Y9G0ZZ, 0H9HXZZ  0JB60ZZ, 0Y910ZZ, 0Y9G30Z, 0H9JX0Z  0JB70ZZ, 0Y9130Z, 0Y9G3ZZ, 0H9JXZZ  0JB80ZZ, 0Y913ZZ, 0Y9G40Z, 0H9KX0Z  0JB90ZZ, 0Y9140Z, 0Y9G4ZZ, 0H9KXZZ  0JBB0ZZ, 0Y914ZZ, 0Y9H00Z, 0H9LX0Z  0JBC0ZZ, 0Y9700Z, 0Y9H0ZZ, 0H9LXZZ  0JBD0ZZ, 0Y970ZZ, 0Y9H30Z, 0H9MX0Z  0JBF0ZZ, 0Y9730Z, 0Y9H3ZZ, 0H9MXZZ  0JBG0ZZ, 0Y973ZZ, 0Y9H40Z, 0H9NX0Z  0JBH0ZZ, 0Y9740Z, 0Y9H4ZZ, 0H9NXZZ  0JBL0ZZ, 0Y974ZZ, 0Y9J00Z, 0J9000Z  0JBM0ZZ, 0Y9800Z, 0Y9J0ZZ, 0J9400Z  0J940ZZ, 0J9D00Z, 0Y9J30Z, 0J9M0ZZ  0J9500Z, 0J9D0ZZ, 0Y9J3ZZ, 0J9N00Z  0J950ZZ, 0J9F00Z, 0Y9J40Z, 0J9N0ZZ  0J9600Z, 0J9F0ZZ, 0Y9J4ZZ, 0J9P00Z  0J960ZZ, 0J9G00Z, 0Y9K00Z, 0J9P0ZZ  0J9700Z, 0J9G0ZZ, 0Y9K0ZZ, 0J9Q00Z  0J970ZZ, 0J9H00Z, 0Y9K30Z, 0J9Q0ZZ  0J9800Z, 0J9H0ZZ, 0Y9K3ZZ, 0J9R00Z  0J980ZZ, 0J9J00Z, 0Y9K40Z, 0J9R0ZZ  0J9900Z, 0J9J0ZZ, 0Y9K4ZZ, 0W9000Z  0J990ZZ, 0J9K00Z, 0Y9L00Z, 0W900ZZ  0J9B00Z, 0J9K0ZZ, 0Y9L0ZZ, 0W9030Z  0J9B0ZZ, 0J9L00Z, 0Y9L30Z, 0W9040Z  0J9C00Z, 0J9L0ZZ, 0Y9L3ZZ, 0W904ZZ  0J9C0ZZ, 0J9M00Z, 0Y9L40Z, 0W9K00Z  0W9L0ZZ, 0W9M0ZZ, 0X920ZZ, 0W9K0ZZ  0W9L30Z, 0W9M30Z, 0X9230Z, 0W9K30Z  0W9L40Z, 0W9M40Z, 0X923ZZ, 0W9K40Z  0W9L4ZZ, 0W9M4ZZ, 0X9240Z, 0W9K4ZZ  0W9M00Z, 0X9200Z, 0W9L00Z,  K650, K51914, L02213, L03322, K651, K5700, L02214, L03323, K6819, K5701, L02215, L03324, K50014, K5720, L02216, L03325, K50114, K5721, L02219, L03326,  K50814, K5740, L03311, L03329, K50914, K5741, L03312, S31020A, K51014, K5780, L03313, S31040A, K51214, K5781, L03314, T8132XA, K51314, K630, L03315, T8131XA, K51414, N99511, L03316, T814XXA, K51514, L02211, L03319, K6811  K51814, L02212, L03321, T8183XA |
| **Laparoscopic Cholecystectomy and Laparoscopic Appendectomy, and related SSI** | |
| 51.23, 51.24, 47.01  0FT44ZZ, 0F544ZZ, 0FB44ZZ, 0DTJ4ZZ | 567, 567.2, 567.21, 567.22, 567.23, 567.29, 567.3, 567.38, 567.39, 567.8, 567.81, 567.89, 567.9, 682.2  K650, K659, L02219, L03319, K651, L02211, L03311, L03321, K652, L02212, L03312, L03322, K6819, L02213, L03313, L03323, K689, L02214, L03314, L03324  K653, L02215, L03315, L03325, K658, L02216, L03316, L03326, L03329 |
| **Orthopedic Procedures and related SSI** | |
| 81.01-81.08, 81.23, 81.24, 81.31-81.38, 81.83, 81.85  0RG0070, 0RGA4JJ, 0RG40KJ, 0SG80JZ, 0RG83J1, 0RQJ4ZZ, 0RGM3KZ  0RG0071, 0RGA4K1, 0RG40Z1, 0SG80KZ, 0RG83JJ, 0RQJXZZ, 0RGM3ZZ  0RG007J, 0RGA4KJ, 0RG40ZJ, 0SG80ZZ, 0RG83K1, 0RQK0ZZ, 0RGM44Z  0RG00A0, 0RGA4Z1, 0RG4371, 0SG834Z, 0RG83KJ, 0RQK3ZZ, 0RGM45Z  0RG00A1, 0RGA4ZJ, 0RG437J, 0SG837Z, 0RG83Z1, 0RQK4ZZ, 0RGM47Z  0RG00AJ, 0SG0070, 0RG43A1, 0SG83JZ, 0RG83ZJ, 0RQKXZZ, 0RGM4JZ  0RG00J0, 0SG00A0, 0RG43AJ, 0SG83KZ, 0RG8471, 0RUE07Z, 0RGM4KZ  0RG00J1, 0SG00J0, 0RG43J1, 0SG83ZZ, 0RG847J, 0RUE0JZ, 0RGM4ZZ  0RG00JJ, 0SG00K0, 0RG43JJ, 0SG844Z, 0RG84A1, 0RUE0KZ, 0RQE0ZZ  0RG00K0, 0SG00Z0, 0RG43K1, 0SG847Z, 0RG84AJ, 0RUE37Z, 0RQE3ZZ  0RG00K1, 0SG0370, 0RG43KJ, 0SG84JZ, 0RG84J1, 0RUE3JZ, 0RQE4ZZ  0RG00KJ, 0SG03A0, 0RG43Z1, 0SG84KZ, 0RG84JJ, 0RUE3KZ, 0RQEXZZ  0RG00Z0, 0SG03J0, 0RG43ZJ, 0SG84ZZ, 0RG84K1, 0RUE47Z, 0RQF0ZZ  0RG00Z1, 0SG03K0, 0RG4471, 0RGE04Z, 0RG84KJ, 0RUE4JZ, 0RQF3ZZ  0RG00ZJ, 0SG03Z0, 0RG447J, 0RGE07Z, 0RG84Z1, 0RUE4KZ, 0RQF4ZZ  0RG0370, 0SG0470, 0RG44A1, 0RGE0JZ, 0RG84ZJ, 0RUF07Z, 0RQFXZZ  0RG0371, 0SG04A0, 0RG44AJ, 0RGE0KZ, 0RGA071, 0RUF0JZ, 0RQG0ZZ  0RG037J, 0SG04J0, 0RG44J1, 0RGE0ZZ, 0RGA07J, 0RUF0KZ, 0RQG3ZZ  0RG03A0, 0SG04K0, 0RG44JJ, 0RGE34Z, 0RGA0A1, 0RUF37Z, 0RQG4ZZ  0RG03A1, 0SG04Z0, 0RG44K1, 0RGE37Z, 0RGA0AJ, 0RUF3JZ, 0RQGXZZ  0RG03AJ, 0SG1070, 0RG44KJ, 0RGE3JZ, 0RGA0J1, 0RUF3KZ, 0RQH0ZZ  0RG03J0, 0SG10A0, 0RG44Z1, 0RGE3KZ, 0RGA0JJ, 0RUF47Z, 0RQH3ZZ  0RG03J1, 0SG10J0, 0RG44ZJ, 0RGE3ZZ, 0RGA0K1, 0RUF4JZ, 0RQH4ZZ  0RG03JJ, 0SG10K0, 0RG6070, 0RGE44Z, 0RGA0KJ, 0RUF4KZ, 0RQHXZZ  0RG03K0, 0SG10Z0, 0RG60A0, 0RGE47Z, 0RGA0Z1, 0RUG07Z, 0RQJ0ZZ  0RG03K1, 0SG1370, 0RG60J0, 0RGE4JZ, 0RGA0ZJ, 0RUG0JZ, 0RQJ3ZZ  0RG03KJ, 0SG13A0, 0RG60K0, 0RGE4KZ, 0RGA371, 0RUG0KZ, 0RG60J1  0RG03Z0, 0SG13J0, 0RG60Z0, 0RGE4ZZ, 0RGA37J, 0RUG37Z, 0RG60JJ  0RG03Z1, 0SG13K0, 0RG6370, 0RGF04Z, 0RGA3A1, 0RUG3JZ, 0RG60K1  0RG03ZJ, 0SG13Z0, 0RG63A0, 0RGF07Z, 0RGA3AJ, 0RUG3KZ, 0RG60KJ  0RG0470, 0SG1470, 0RG63J0, 0RGF0JZ, 0RGA3J1, 0RUG47Z, 0RG60Z1  0RG0471, 0SG14A0, 0RG63K0, 0RGF0KZ, 0RGA3JJ, 0RUG4JZ, 0RG60ZJ  0RG047J, 0SG14J0, 0RG63Z0, 0RGF0ZZ, 0RGA3K1, 0RUG4KZ, 0RG6371  0RG04A0, 0SG14K0, 0RG6470, 0RGF34Z, 0RGA3KJ, 0RUH07Z, 0RG637J  0RG04A1, 0SG14Z0, 0RG64A0, 0RGF37Z, 0RGA3Z1, 0RUH0JZ, 0RG63A1  0RG04AJ, 0SG3070, 0RG64J0, 0RGF3JZ, 0RGA3ZJ, 0RUH0KZ, 0RG63AJ  0RG04J0, 0SG30A0, 0RG64K0, 0RGF3KZ, 0RGA471, 0RUH37Z, 0RG63J1  0RG04J1, 0SG30J0, 0RG64Z0, 0RGF3ZZ, 0RGA47J, 0RUH3JZ, 0RG63JJ  0RG04JJ, 0SG30K0, 0RG7070, 0RGF44Z, 0RGA4A1, 0RUH3KZ, 0RG63K1  0RG04K0, 0SG30Z0, 0RG70A0, 0RGF47Z, 0RGA4AJ, 0RUH47Z, 0RG63KJ  0RG04K1, 0SG3370, 0RG70J0, 0RGF4JZ, 0RGA4J1, 0RUH4JZ, 0RG63Z1  0RG04KJ, 0SG33A0, 0RG70K0, 0RGF4KZ, 0RGJ4KZ, 0RUH4KZ, 0RG63ZJ  0RG04Z0, 0SG33J0, 0RG70Z0, 0RGF4ZZ, 0RGJ4ZZ, 0RUJ07Z, 0RG6471  0RG04Z1, 0SG33K0, 0RG7370, 0RGG04Z, 0RGK04Z, 0RUJ0JZ, 0RG647J  0RG04ZJ, 0SG33Z0, 0RG73A0, 0RGG07Z, 0RGK07Z, 0RUJ0KZ, 0RG64A1  0RG1070, 0SG3470, 0RG73J0, 0RGG0JZ, 0RGK0JZ, 0RUJ37Z, 0RG64AJ  0RG10A0, 0SG34A0, 0RG73K0, 0RGG0KZ, 0RGK0KZ, 0RUJ3JZ, 0RG64J1  0RG10J0, 0SG34J0, 0RG73Z0, 0RGG0ZZ, 0RGK0ZZ, 0RUJ3KZ, 0RG64JJ  0RG10K0, 0SG34K0, 0RG7470, 0RGG34Z, 0RGK34Z, 0RUJ47Z, 0RG64K1  0RG10Z0, 0SG34Z0, 0RG74A0, 0RGG37Z, 0RGK37Z, 0RUJ4JZ, 0RG64KJ  0RG1370, 0SG0071, 0RG74J0, 0RGG3JZ, 0RGK3JZ, 0RUJ4KZ, 0RG64Z1  0RG13A0, 0SG00A1, 0RG74K0, 0RGG3KZ, 0RGK3KZ, 0RUK07Z, 0RG64ZJ  0RG13J0, 0SG00J1, 0RG74Z0, 0RGG3ZZ, 0RGK3ZZ, 0RUK0JZ, 0RG7071  0RG13K0, 0SG00K1, 0RG8070, 0RGG44Z, 0RGK44Z, 0RUK0KZ, 0RG707J  0RG13Z0, 0SG00Z1, 0RG80A0, 0RGG47Z, 0RGK47Z, 0RUK37Z, 0RG70A1  0RG1470, 0SG0371, 0RG80J0, 0RGG4JZ, 0RGK4JZ, 0RUK3JZ, 0RG70AJ  0RG14A0, 0SG03A1, 0RG80K0, 0RGG4KZ, 0RGK4KZ, 0RUK3KZ, 0RG70J1  0RG14J0, 0SG03J1, 0RG80Z0, 0RGG4ZZ, 0RGK4ZZ, 0RUK47Z, 0RG70JJ  0RG14K0, 0SG03K1, 0RG8370, 0RGH04Z, 0RGL04Z, 0RUK4JZ, 0RG70K1  0RG14Z0, 0SG03Z1, 0RG83A0, 0RGH07Z, 0RGL05Z, 0RUK4KZ, 0RG70KJ  0RG2070, 0SG0471, 0RG83J0, 0RGH0JZ, 0RGL07Z, 0RQL0ZZ, 0RG70Z1  0RG20A0, 0SG04A1, 0RG83K0, 0RGH0KZ, 0RGL0JZ, 0RQL3ZZ, 0RG70ZJ  0RG20J0, 0SG04J1, 0RG83Z0, 0RGH0ZZ, 0RGL0KZ, 0RQL4ZZ, 0RG7371  0RG20K0, 0SG04K1, 0RG8470, 0RGH34Z, 0RGL0ZZ, 0RQLXZZ, 0RG737J  0RG20Z0, 0SG04Z1, 0RG84A0, 0RGH37Z, 0RGL34Z, 0RQM0ZZ, 0RG73A1  0RG20ZJ, 0SG1071, 0RG84J0, 0RGH3JZ, 0RGL35Z, 0RQM3ZZ, 0RG73AJ  0RG2370, 0SG10A1, 0RG84K0, 0RGH3KZ, 0RGL37Z, 0RQM4ZZ, 0RG73J1  0RG23A0, 0SG10J1, 0RG84Z0, 0RGH3ZZ, 0RGL3JZ, 0RQMXZZ, 0RG73JJ  0RG23J0, 0SG10K1, 0RGA070, 0RGH44Z, 0RGL3KZ, 0RUL07Z, 0RG73K1  0RG23K0, 0SG10Z1, 0RGA0A0, 0RGH47Z, 0RGL3ZZ, 0RUL0JZ, 0RG73KJ  0RG23Z0, 0SG1371, 0RGA0J0, 0RGH4JZ, 0RGL44Z, 0RUL0KZ, 0RG73Z1  0RG2470, 0SG13A1, 0RGA0K0, 0RGH4KZ, 0RGL45Z, 0RUL37Z, 0RG73ZJ  0RG24A0, 0SG13J1, 0RGA0Z0, 0RGH4ZZ, 0RGL47Z, 0RUL3JZ, 0RG7471  0RG24J0, 0SG13K1, 0RGA370, 0RGJ04Z, 0RGL4JZ, 0RUL3KZ, 0RG747J  0RG24K0, 0SG13Z1, 0RGA3A0, 0RGJ07Z, 0RGL4KZ, 0RUL47Z, 0RG74A1  0RG24Z0, 0SG1471, 0RGA3J0, 0RGJ0JZ, 0RGL4ZZ, 0RUL4JZ, 0RG74AJ  0RG4070, 0SG14A1, 0RGA3K0, 0RGJ0KZ, 0RGM04Z, 0RUL4KZ, 0RG74J1  0RG40A0, 0SG14J1, 0RGA3Z0, 0RGJ0ZZ, 0RGM05Z, 0RUM07Z, 0RG74JJ  0RG40J0, 0SG14K1, 0RGA470, 0RGJ34Z, 0RGM07Z, 0RUM0JZ, 0RG74K1  0RG40K0, 0SG14Z1, 0RGA4A0, 0RGJ37Z, 0RGM0JZ, 0RUM0KZ, 0RG74KJ  0RG40Z0, 0SG3071, 0RGA4J0, 0RGJ3JZ, 0RGM0KZ, 0RUM37Z, 0RG74Z1  0RG4370, 0SG30A1, 0RGA4K0, 0RGJ3KZ, 0RGM0ZZ, 0RUM3JZ, 0RG74ZJ  0RG43A0, 0SG30J1, 0RGA4Z0, 0RGJ3ZZ, 0RGM34Z, 0RUM3KZ, 0RG8071  0RG43J0, 0SG30K1, 0RG6071, 0RGJ44Z, 0RGM35Z, 0RUM47Z, 0RG807J  0RG43K0, 0SG30Z1, 0RG607J, 0RGJ47Z, 0RGM37Z, 0RUM4JZ, 0RG80A1  0RG43Z0, 0SG3371, 0RG60A1, 0RGJ4JZ, 0RGM3JZ, 0RUM4KZ, 0RG80AJ  0RG4470, 0SG33A1, 0RG60AJ, 0RG20A1, 0SG14KJ, 0RG83AJ, 0RG80J1  0RG44A0, 0SG33J1, 0RG20AJ, 0SG14ZJ, 0RG14ZJ, 0SG147J, 0RG80JJ  0RG44J0, 0SG33K1, 0RG20J1, 0SG307J, 0RG2071, 0SG14AJ, 0RG80K1  0RG44K0, 0SG33Z1, 0RG20JJ, 0SG30AJ, 0RG207J, 0SG14JJ, 0RG80KJ  0RG44Z0, 0SG3471, 0RG20K1, 0SG30JJ, 0RG1471, 0SG10AJ, 0RG80Z1  0RG1071, 0SG34A1, 0RG20KJ, 0SG30KJ, 0RG147J, 0SG10JJ, 0RG80ZJ  0RG107J, 0SG34J1, 0RG20Z1, 0SG30ZJ, 0RG14A1, 0SG10KJ, 0RG8371  0RG10A1, 0SG34K1, 0RG2371, 0SG337J, 0RG14AJ, 0SG10ZJ, 0RG837J  0RG10AJ, 0SG34Z1, 0RG237J, 0SG33AJ, 0RG14J1, 0SG137J, 0RG83A1  0RG10J1, 0SG007J, 0RG23A1, 0SG33JJ, 0RG14JJ, 0SG13AJ, 0SG73JZ  0RG10JJ, 0SG00AJ, 0RG23AJ, 0SG33KJ, 0RG14K1, 0SG13JJ, 0SG73KZ  0RG10K1, 0SG00JJ, 0RG23J1, 0SG33ZJ, 0RG14KJ, 0SG13KJ, 0SG73ZZ  0RG10KJ, 0SG00KJ, 0RG23JJ, 0SG347J, 0RG14Z1, 0SG13ZJ, 0SG744Z  0RG10Z1, 0SG00ZJ, 0RG23K1, 0SG34AJ, 0RG407J, 0SG747Z, 0RG24KJ  0RG10ZJ, 0SG037J, 0RG23KJ, 0SG34JJ, 0RG40A1, 0SG74JZ, 0RG24Z1  0RG1371, 0SG03AJ, 0RG23Z1, 0SG34KJ, 0RG40AJ, 0SG74KZ, 0RG24ZJ  0RG137J, 0SG03JJ, 0RG23ZJ, 0SG34ZJ, 0RG40J1, 0SG74ZZ, 0RG4071  0RG13A1, 0SG03KJ, 0RG2471, 0SG704Z, 0RG40JJ, 0SG804Z, 0SG04KJ  0RG13AJ, 0SG03ZJ, 0RG247J, 0SG707Z, 0RG40K1, 0SG807Z, 0SG04ZJ  0RG13J1, 0SG047J, 0RG24A1, 0SG70JZ, 0RG24JJ, 0SG734Z, 0SG107J  0RG13JJ, 0SG04AJ, 0RG24AJ, 0SG70KZ, 0RG24K1, 0SG737Z, 0RG13Z1  0RG13K1, 0SG04JJ, 0RG24J1, 0SG70ZZ, 0RG13KJ, 0RG13ZJ | 996.67, 998.59  T8460XA, T84613A, T84620A, T84624A, T8469XA, T84610A, T84614A, T84621A, T84625A, T847XXA, T84611A, T84615A, T84622A, T84629A, K6811, T84612A, T84619A, T84623A, T8463XA, T814XXA |
| **Cardiac Implantable Electronic Device and related SSI** | |
| 00.50, 00.51, 00.52, 00.53, 00.54, 37.80, 37.81, 37.82, 37.83, 37.85, 37.86, 37.87, 37.94, 37.96, 37.98, 37.74, 37.75, 37.76, 37.77, 37.79, 37.89  02H40JZ, 0JH804Z, 02HL3KZ, 0JH838Z, 0JPT3PZ, 02H73MZ, 02HK3KZ  02H43JZ, 0JH834Z, 02HL4KZ, 02HN0JZ, 0JH60PZ, 02HK3MZ, 02HK4KZ  02H44JZ, 0JH605Z, 0JH609Z, 02HN0MZ, 0JH63PZ, 02HL3MZ, 02HL0KZ  02H60JZ, 0JH635Z, 0JH639Z, 02HN3JZ, 0JH80PZ, 0JWT0PZ, 02H74KZ  02H63JZ, 0JH805Z, 0JH809Z, 02HN3MZ, 0JH83PZ, 0JWT3PZ, 02PAXMZ  02H64JZ, 0JH835Z, 0JH839Z, 02HN4JZ, 0JH604Z, 0JH607Z, 0JH608Z  02HK0JZ, 0JH606Z, 02H43KZ, 02HN4MZ, 0JH634Z, 0JH637Z, 0JH638Z  02HK3JZ, 0JH636Z, 02H43MZ, 02WA0MZ, 02H64KZ, 0JH807Z, 0JH808Z  02HK4JZ, 0JH806Z, 02PA0MZ, 02WA3MZ, 02H70KZ, 0JH837Z, 02H63MZ  02HL0JZ, 0JH836Z, 02PA3MZ, 02WA4MZ, 02H73KZ, 02HK0KZ, 02H73JZ  02HL3JZ, 02H60KZ, 02PA4MZ, 02HL4JZ, 02H63KZ, 0JPT0PZ | 996.61, 998.59  T826XXA, T827XXA, K6811, T814XXA |
| **All other procedures and related SSI** | |
| ICD 9 and ICD 10 codes for all other procedures are based on the procedure codes from AHRQ, SAS PSI Software, Version 5.0.3 (ICD 9 codes), and Version 6.0.1 (ICD 10 codes). They are too long to list here. Both can be downloaded at: <https://qualityindicators.ahrq.gov/archive/software> | 998.5, 998.51, 998.59, 996.69, 567.2–567.29, 567.9, 567.3–567.39, 682.2, 682.9  T814XXA, K6812, L02219, L03322, K6811, K6819, L03311, L03323, T8572XA, K689, L03312, L03324, T8579XA, L02211, L03313, L03325, T86842, L02212, L03314, L03326, K650, L02213, L03315, L03329  K651, L02214, L03316, L0291, K652, L02215, L03319, L0390, K659, L02216, L03321, L0391, L983 |
|  | **Alternative general SSI** |
|  | 998.5, 998.51, 998.59, 996.6-996.69  T814XXA, T836XXA, T8460XA, T84619A, T84629A, K6811, T8450XA, T84610A, T84620A, T8463XA, T8579XA, T8451XA, T84611A, T84621A, T8469XA, T826XXA, T8452XA, T84612A, T84622A, T847XXA  T827XXA, T8453XA, T84613A, T84623A, T8571XA, T8351XA, T8454XA, T84614A, T84624A, T8572XA, T8359XA, T8459XA, T84615A, T84625A, T86842 |
