# Supplementary material for: Evaluation of hospital-acquired conditions reduction program in surgical procedures
Source: PLoS One. 2025 Nov 21;20(11):e0337072. doi: 10.1371/journal.pone.0337072 (PMC12637954; doi:10.1371/journal.pone.0337072)
Supplement: S2 Table — Procedure rates are the percentage of total discharges that underwent the corresponding procedures. SSI rates are the number of infections per 1,000 discharges for each procedure. (DOCX) [file pone.0337072.s003.docx]

**S2 Table Procedure and SSI Rates by Admission Year and Quarter**

|  | 2012Q1 | 2012Q2 | 2012Q3 | 2012Q4 | 2013Q1 | 2013Q2 | 2013Q3 | 2013Q4 | 2014Q1 | 2014Q2 | 2014Q3 | 2014Q4 | 2015Q1 | 2015Q2 | 2015Q3 | Total |
| --- | --- | --- | --- | --- | --- | --- | --- | --- | --- | --- | --- | --- | --- | --- | --- | --- |
| N | 601,548 | 563,766 | 548,916 | 567,750 | 594,144 | 557,849 | 540,416 | 543,230 | 557,019 | 549,917 | 534,802 | 555,076 | 590,968 | 562,053 | 512,479 | 8,379,933 |
| **Panel A. Percentage of discharges that underwent different procedures (%)** | | | | | | | | | | | | | | | | |
| **Abdominal hysterectomy^a^** | | | | | | | | | | | | | | | | |
|  | 0.16 | 0.17 | 0.17 | 0.16 | 0.14 | 0.15 | 0.16 | 0.15 | 0.13 | 0.14 | 0.14 | 0.13 | 0.13 | 0.14 | 0.14 | 0.15 |
| **Colon surgery^c^** | | | | | | | | | | | | | | | | |
|  | 0.97 | 0.99 | 1.01 | 0.98 | 0.90 | 0.98 | 1.03 | 0.97 | 0.95 | 0.95 | 0.97 | 0.89 | 0.90 | 0.96 | 0.94 | 0.96 |
| **Laparoscopic cholecystectomy and laparoscopic appendectomy^e^** | | | | | | | | | | | | | | | | |
|  | 0.73 | 0.78 | 0.84 | 0.75 | 0.68 | 0.78 | 0.82 | 0.75 | 0.73 | 0.75 | 0.79 | 0.72 | 0.74 | 0.80 | 0.85 | 0.77 |
| **Orthopedic procedures^g^** | | | | | | | | | | | | | | | | |
|  | 0.92 | 0.97 | 1.00 | 0.98 | 0.95 | 1.03 | 1.09 | 1.07 | 1.07 | 1.12 | 1.19 | 1.09 | 1.15 | 1.21 | 1.30 | 1.07 |
| **Cardiac implantable electronic device^i^** | | | | | | | | | | | | | | | | |
|  | 0.74 | 0.74 | 0.73 | 0.67 | 0.83 | 0.94 | 0.95 | 0.84 | 0.85 | 0.91 | 0.91 | 0.75 | 0.88 | 0.94 | 0.97 | 0.84 |
| **All other procedure^k^** | | | | | | | | | | | | | | | | |
|  | 24.87 | 25.52 | 25.68 | 24.77 | 24.53 | 26.03 | 26.67 | 25.85 | 25.77 | 25.95 | 26.58 | 25.01 | 24.61 | 25.84 | 26.42 | 25.58 |
| **Panel B. SSI Rates for different procedures (number of infections per 1,000 discharges)** | | | | | | | | | | | | | | | | |
| **SSI rates among abdominal hysterectomy^b^** | | | | | | | | | | | | | | | | |
|  | 17.19 | 13.73 | 19.15 | 23.63 | 22.25 | 10.66 | 18.82 | 17.63 | 22.5 | 16.06 | 16.39 | 19.86 | 24.08 | 10.31 | 15.41 | 17.84 |
| **SSI rates among colon surgery^d^** | | | | | | | | | | | | | | | | |
|  | 156.49 | 162.17 | 155.16 | 157.76 | 162.69 | 153.52 | 153.31 | 158.66 | 151.68 | 163.28 | 154.89 | 165.56 | 161.65 | 160.02 | 152.97 | 157.96 |
| **SSI rates among laparoscopic cholecystectomy and laparoscopic appendectomy^f^** | | | | | | | | | | | | | | | | |
|  | 11.91 | 12.01 | 12.98 | 14.12 | 15.16 | 13.07 | 11.53 | 16.22 | 15.24 | 15.02 | 15.14 | 15.79 | 16.4 | 13.56 | 14.65 | 14.14 |
| **SSI rates among orthopedic procedures^h^** | | | | | | | | | | | | | | | | |
|  | 3.79 | 3.67 | 4.19 | 5.02 | 2.31 | 4.36 | 5.08 | 2.93 | 3.36 | 3.1 | 2.84 | 1.82 | 2.35 | 4.13 | 3.45 | 3.47 |
| **SSI rates among cardiac implantable electronic device^j^** | | | | | | | | | | | | | | | | |
|  | 42.71 | 47.53 | 44.86 | 56.14 | 44.2 | 40.15 | 39.88 | 41.46 | 39.1 | 35.84 | 51.13 | 47.08 | 33.03 | 38.84 | 45.3 | 42.74 |
| **SSI rates among all other procedure^l^** | | | | | | | | | | | | | | | | |
|  | 23.93 | 25.3 | 26.34 | 24.79 | 25.36 | 24.57 | 26.03 | 26.43 | 25.29 | 25.43 | 26.44 | 25.11 | 24.69 | 24.84 | 22.51 | 25.14 |
| ^a^ Percentage of patients with abdominal hysterectomy if their first, i.e., primary, ICD-9 procedure codes were 68.31, 68.39, 68.41, 68.49, 68.61, and 68.69. ^b^ SSIs are identified by ICD-9 codes: 567.22, 682.2, 998.31, 998.32, 998.51, and 998.59, including the first diagnoses. ^c^ Percentage of patients that had colon surgeries if their first, i.e., primary, ICD-9 procedure codes were 17.31-17.36, 17.39, 45.03, 45.26, 45.41, 45.49, 45.52, 45.71-45.76, 45.79, 45.81-45.83, 45.92-45.95, 46.03, 46.04, 46.10, 46.11, 46.13, 46.14, 46.43, 46.52, 46.75, 46.76, and 46.94. ^d^ SSI for colon surgeries 567.21, 567.22, 567.29, 567.38, 569.5, 596.61, 596.81, 682.2, 879.9, 998.31, 998.32, 998.51, 998.59, 998.6, 54.0, 54.11, 54.19, 86.04, 86.22, and 86.28, including the first diagnoses.  ^e^ Laparoscopic cholecystectomy and laparoscopic appendectomy (procedure codes: 51.23, 51.24, 47.01) ^f^ SSI: 567, 567.2, 567.21, 567.22, 567.23, 567.29, 567.3, 567.38, 567.39, 567.8, 567.81, 567.89, 567.9, 682.2 ^g^ Orthopedic Procedures (procedure codes: 81.01-81.08, 81.23, 81.24, 81.31-81.38, 81.83, 81.85.^h^ SSI: 996.67, 998.59 ^i^ Cardiac Implantable Electronic Device (procedure codes: 00.50, 00.51, 00.52, 00.53, 00.54, 37.80, 37.81, 37.82, 37.83, 37.85, 37.86, 37.87, 37.94, 37.96, 37.98, 37.74, 37.75, 37.76, 37.77, 37.79, 37.89. ^j^ SSI: 996.61, 998.59 ^k^surgical procedures other than abdominal hysterectomy and colon procedures ^l^ SSI: 998.5, 998.51, 998.59, 996.6-996.69 | | | | | | | | | | | | | | | | |
