# Supplementary material for: Evaluation of hospital-acquired conditions reduction program in surgical procedures
Source: PLoS One. 2025 Nov 21;20(11):e0337072. doi: 10.1371/journal.pone.0337072 (PMC12637954; doi:10.1371/journal.pone.0337072)
Supplement: S3 Table — (DOCX) [file pone.0337072.s004.docx]

S3 Table includes two panels for summary statistics when alternative procedures and SSI definitions are used. Procedure rates are the percentage of total discharges that underwent the corresponding procedures. SSI rates are the number of infections per 1,000 discharges for each procedure.

Panel A of S3 Table shows SSI rates among procedures defined by each discharge’s first, i.e., primary, ICD-9 procedure codes. Panel B is based on all procedures, including the primary. If relevant codes were ever reported in the discharge, the corresponding procedures were identified. The detailed procedure codes and SSI codes are in the “Treatment and Control Outcome Variables” and “Sensitivity Analysis” sections in the manuscript. They are also included in S3 Table’s footnote. Although the exact numbers differ, the fluctuation patterns are similar despite alternative procedures and SSI definitions.

**S3 Table. Procedure and SSI Rates by Alternative Procedures and Definitions by Admission Year and Quarter**

|  | **Panel A: Procedures Defined by Primary Diagnoses** | | | | | | | | | | | | | | | |
| --- | --- | --- | --- | --- | --- | --- | --- | --- | --- | --- | --- | --- | --- | --- | --- | --- |
|  | 2012Q1 | 2012Q2 | 2012Q3 | 2012Q4 | 2013Q1 | 2013Q2 | 2013Q3 | 2013Q4 | 2014Q1 | 2014Q2 | 2014Q3 | 2014Q4 | 2015Q1 | 2015Q2 | 2015Q3 | Total |
| N | 601,548 | 563,766 | 548,916 | 567,750 | 594,144 | 557,849 | 540,416 | 543,230 | 557,019 | 549,917 | 534,802 | 555,076 | 590,968 | 562,053 | 512,479 | 8,379,933 |
| **Abdominal hysterectomy^a^ (% of discharges that underwent this procedures)** | | | | | | | | | | | | | | | | |
|  | 0.164 | 0.168 | 0.171 | 0.164 | 0.136 | 0.151 | 0.157 | 0.146 | 0.128 | 0.136 | 0.137 | 0.127 | 0.134 | 0.138 | 0.139 | 0.147 |
| SSI first definition (start from second diagnoses)^b^ (number of infections per 1,000 discharges) | | | | | | | | | |  |  |  |  |  |  |  |
|  | 17.19 | 13.73 | 19.15 | 23.63 | 21.01 | 10.66 | 18.82 | 17.63 | 22.5 | 16.06 | 16.39 | 19.86 | 22.81 | 10.31 | 15.41 | 17.67 |
| SSI first definition (all diagnoses)^c^ | | | | | |  |  |  |  |  |  |  |  |  |  |  |
|  | 17.19 | 13.73 | 19.15 | 23.63 | 22.25 | 10.66 | 18.82 | 17.63 | 22.5 | 16.06 | 16.39 | 19.86 | 24.08 | 10.31 | 15.41 | 17.84 |
| SSI second definition (start from second diagnoses)^d^ | | | | | |  |  |  |  |  |  |  |  |  |  |  |
|  | 16.18 | 15.84 | 21.28 | 21.48 | 19.78 | 9.48 | 12.94 | 18.89 | 22.5 | 18.74 | 19.13 | 19.86 | 16.48 | 12.89 | 14.01 | 17.27 |
| SSI second definition (all diagnoses)^e^ | | | | | |  |  |  |  |  |  |  |  |  |  |  |
|  | 16.18 | 15.84 | 21.28 | 21.48 | 19.78 | 9.48 | 12.94 | 18.89 | 22.5 | 18.74 | 19.13 | 19.86 | 17.74 | 12.89 | 14.01 | 17.35 |
| SSI third definition (start from second diagnoses)^f^ | | | | | |  |  |  |  |  |  |  |  |  |  |  |
|  | 12.13 | 11.62 | 13.83 | 15.04 | 14.83 | 7.11 | 10.59 | 13.85 | 18.28 | 12.05 | 13.66 | 12.77 | 13.94 | 7.73 | 12.61 | 12.62 |
| SSI third definition (all diagnoses)^g^ | | | | | |  |  |  |  |  |  |  |  |  |  |  |
|  | 12.13 | 11.62 | 13.83 | 15.04 | 16.07 | 7.11 | 10.59 | 13.85 | 18.28 | 12.05 | 13.66 | 12.77 | 13.94 | 7.73 | 12.61 | 12.71 |
| **Colon surgery (% of discharges that underwent this procedures)** | | | | | | | | | | | | | | | | |
|  | 0.969 | 0.986 | 1.011 | 0.976 | 0.902 | 0.982 | 1.030 | 0.973 | 0.948 | 0.950 | 0.968 | 0.893 | 0.904 | 0.964 | 0.943 | 0.959 |
| SSI first definition (start from second diagnoses)^h^ (number of infections per 1,000 discharges) | | | | | | | | | |  |  |  |  |  |  |  |
|  | 151.68 | 158.93 | 152.1 | 153.97 | 158.96 | 149.69 | 150.43 | 155.45 | 147.32 | 160.03 | 152.95 | 162.73 | 159.4 | 154.85 | 149.87 | 154.52 |
| SSI first definition (all diagnoses)^i^ | | | | | |  |  |  |  |  |  |  |  |  |  |  |
|  | 156.49 | 162.17 | 155.16 | 157.76 | 162.69 | 153.52 | 153.31 | 158.66 | 151.68 | 163.28 | 154.89 | 165.56 | 161.65 | 160.02 | 152.97 | 157.96 |
| SSI second definition (start from second diagnoses) | | | | | |  |  |  |  |  |  |  |  |  |  |  |
|  | 115.31 | 121.67 | 123.45 | 120.76 | 126.49 | 117.74 | 120.6 | 127.46 | 120.05 | 123.66 | 124.76 | 129.86 | 131.52 | 122 | 109.29 | 122.29 |
| SSI second definition (all diagnoses) | | | | | |  |  |  |  |  |  |  |  |  |  |  |
|  | 117.88 | 124.73 | 125.79 | 122.92 | 129.85 | 120.12 | 122.39 | 129.16 | 122.7 | 126.91 | 126.3 | 131.88 | 133.02 | 124.95 | 111.36 | 124.65 |
| SSI third definition (start from second diagnoses) | | | | | |  |  |  |  |  |  |  |  |  |  |  |
|  | 44.27 | 41.22 | 43.61 | 42.06 | 40.67 | 35.41 | 42.24 | 41.04 | 42.23 | 37.52 | 39.01 | 38.11 | 35.73 | 33.04 | 30.22 | 39.2 |
| SSI third definition (all diagnoses) | | | | | |  |  |  |  |  |  |  |  |  |  |  |
|  | 46.33 | 43.74 | 46.31 | 44.77 | 44.03 | 37.24 | 45.29 | 44.25 | 44.5 | 40.39 | 41.71 | 41.94 | 38.91 | 35.07 | 33.74 | 41.98 |
| **Cardiac implantable electronic device (% of discharges that underwent this procedures)** | | | | | | | | | | | | | | | | |
|  | 0.740 | 0.735 | 0.727 | 0.665 | 0.826 | 0.942 | 0.946 | 0.835 | 0.845 | 0.913 | 0.907 | 0.750 | 0.881 | 0.944 | 0.965 | 0.840 |
| SSI first definition (start from second diagnoses)^l^ (number of infections per 1,000 discharges) | | | | | | | | | |  |  |  |  |  |  |  |
|  | 5.62 | 6.51 | 4.76 | 5.56 | 6.93 | 4.76 | 3.71 | 5.07 | 4.46 | 3.78 | 3.71 | 5.28 | 3.65 | 5.84 | 5.86 | 5 |
| SSI first definition (all diagnoses)^m^ | | | | | |  |  |  |  |  |  |  |  |  |  |  |
|  | 42.71 | 47.53 | 44.86 | 56.14 | 44.2 | 40.15 | 39.88 | 41.46 | 39.1 | 35.84 | 51.13 | 47.08 | 33.03 | 38.84 | 45.3 | 42.74 |
| SSI second definition (start from second diagnoses) | | | | | |  |  |  |  |  |  |  |  |  |  |  |
|  | 5.39 | 7.96 | 8.27 | 9 | 9.17 | 6.28 | 6.65 | 7.06 | 8.07 | 7.57 | 11.13 | 9.13 | 6.34 | 7.92 | 5.86 | 7.67 |
| SSI second definition (all diagnoses) | | | | | |  |  |  |  |  |  |  |  |  |  |  |
|  | 5.62 | 8.44 | 9.27 | 10.06 | 9.37 | 6.66 | 7.04 | 8.16 | 8.71 | 7.96 | 11.55 | 9.61 | 6.72 | 8.3 | 6.47 | 8.2 |
| SSI third definition (start from second diagnoses) | | | | | |  |  |  |  |  |  |  |  |  |  |  |
|  | 7.19 | 8.44 | 7.52 | 7.42 | 10.8 | 7.61 | 4.89 | 6.39 | 5.74 | 5.77 | 7.01 | 6.73 | 6.34 | 7.35 | 7.28 | 7.08 |
| SSI third definition (all diagnoses) | | | | | |  |  |  |  |  |  |  |  |  |  |  |
|  | 44.5 | 49.46 | 48.12 | 58.53 | 48.07 | 42.82 | 41.06 | 42.56 | 40.59 | 38.03 | 54.23 | 48.52 | 35.91 | 40.72 | 47.12 | 44.95 |
| **Laparoscopic cholecystectomy and laparoscopic appendectomy (% of discharges that underwent this procedures)** | | | | | | | | | | | | | | | | |
|  | 0.726 | 0.783 | 0.842 | 0.749 | 0.677 | 0.782 | 0.819 | 0.749 | 0.730 | 0.751 | 0.790 | 0.719 | 0.743 | 0.800 | 0.853 | 0.766 |
| SSI first definition (start from second diagnoses)^j^ (number of infections per 1,000 discharges) | | | | | | | | | |  |  |  |  |  |  |  |
|  | 11.68 | 12.01 | 12.98 | 13.88 | 15.16 | 13.07 | 11.08 | 15.72 | 15.24 | 14.77 | 15.14 | 15.79 | 16.4 | 13.56 | 14.65 | 14.03 |
| SSI first definition (all diagnoses)^k^ | | | | | |  |  |  |  |  |  |  |  |  |  |  |
|  | 11.91 | 12.01 | 12.98 | 14.12 | 15.16 | 13.07 | 11.53 | 16.22 | 15.24 | 15.02 | 15.14 | 15.79 | 16.4 | 13.56 | 14.65 | 14.14 |
| SSI second definition (start from second diagnoses) | | | | | |  |  |  |  |  |  |  |  |  |  |  |
|  | 12.83 | 10.87 | 14.06 | 14.59 | 15.91 | 12.84 | 11.53 | 17.69 | 13.77 | 13.32 | 15.85 | 16.55 | 16.17 | 13.78 | 15.79 | 14.33 |
| SSI second definition (all diagnoses) | | | | | |  |  |  |  |  |  |  |  |  |  |  |
|  | 13.29 | 10.87 | 14.28 | 14.82 | 16.16 | 12.84 | 12.66 | 18.43 | 14.26 | 13.8 | 16.56 | 16.8 | 16.4 | 14.23 | 16.02 | 14.72 |
| SSI third definition (start from second diagnoses) | | | | | |  |  |  |  |  |  |  |  |  |  |  |
|  | 5.73 | 4.76 | 6.71 | 6.12 | 5.72 | 7.8 | 5.2 | 6.88 | 4.92 | 5.33 | 6.63 | 7.52 | 6.38 | 6.22 | 7.09 | 6.2 |
| SSI third definition (all diagnoses) | | | | | |  |  |  |  |  |  |  |  |  |  |  |
|  | 6.41 | 4.98 | 6.92 | 6.82 | 6.21 | 8.03 | 6.33 | 7.62 | 5.41 | 5.81 | 8.52 | 8.27 | 6.83 | 6.89 | 7.55 | 6.84 |
| **Orthopedic procedures (% of discharges that underwent this procedures)** | | | | | | | | | | | | | | | | |
|  | 0.921 | 0.966 | 1.001 | 0.982 | 0.945 | 1.028 | 1.092 | 1.068 | 1.069 | 1.115 | 1.185 | 1.086 | 1.154 | 1.206 | 1.302 | 1.072 |
| SSI first definition (start from second diagnoses)^l^ (number of infections per 1,000 discharges) | | | | | | | | | |  |  |  |  |  |  |  |
|  | 2.71 | 1.84 | 2.73 | 3.77 | 1.78 | 2.62 | 3.56 | 1.72 | 2.01 | 1.96 | 2.05 | 1.33 | 1.61 | 3.1 | 2.1 | 2.32 |
| SSI first definition (all diagnoses)^m^ | | | | | |  |  |  |  |  |  |  |  |  |  |  |
|  | 3.79 | 3.67 | 4.19 | 5.02 | 2.31 | 4.36 | 5.08 | 2.93 | 3.36 | 3.1 | 2.84 | 1.82 | 2.35 | 4.13 | 3.45 | 3.47 |
| SSI second definition (start from second diagnoses) | | | | | |  |  |  |  |  |  |  |  |  |  |  |
|  | 4.15 | 2.57 | 4.19 | 3.77 | 2.85 | 4.18 | 5.08 | 2.58 | 2.35 | 2.28 | 3.94 | 2.99 | 2.49 | 3.54 | 2.4 | 3.27 |
| SSI second definition (all diagnoses) | | | | | |  |  |  |  |  |  |  |  |  |  |  |
|  | 4.69 | 3.31 | 4.73 | 4.66 | 3.2 | 4.71 | 5.59 | 2.76 | 2.85 | 3.26 | 4.42 | 3.48 | 2.79 | 3.84 | 3.15 | 3.81 |
| SSI third definition (start from second diagnoses) | | | | | |  |  |  |  |  |  |  |  |  |  |  |
|  | 5.05 | 3.12 | 5.1 | 4.48 | 3.03 | 3.31 | 5.25 | 2.58 | 3.69 | 3.42 | 4.26 | 2.65 | 2.79 | 5.46 | 4.79 | 3.94 |
| SSI third definition (all diagnoses) | | | | | |  |  |  |  |  |  |  |  |  |  |  |
|  | 5.96 | 5.14 | 6.55 | 5.74 | 3.56 | 5.23 | 6.78 | 4.14 | 5.04 | 4.57 | 5.05 | 3.32 | 3.52 | 6.49 | 6.14 | 5.14 |
|  | **Panel B: Procedures Defined by All Diagnose** | | | | | | | | | | | | | | | |
|  | 2012Q1 | 2012Q2 | 2012Q3 | 2012Q4 | 2013Q1 | 2013Q2 | 2013Q3 | 2013Q4 | 2014Q1 | 2014Q2 | 2014Q3 | 2014Q4 | 2015Q1 | 2015Q2 | 2015Q3 | Total |
|  | 601,548 | 563,766 | 548,916 | 567,750 | 594,144 | 557,849 | 540,416 | 543,230 | 557,019 | 549,917 | 534,802 | 555,076 | 590,968 | 562,053 | 512,479 | 8,379,933 |
| **Abdominal hysterectomy^a^ (% of discharges that underwent this procedures)** | | | | | | | | | | | | | | | | |
|  | 0.209 | 0.215 | 0.217 | 0.212 | 0.190 | 0.213 | 0.227 | 0.213 | 0.186 | 0.200 | 0.211 | 0.180 | 0.179 | 0.190 | 0.196 | 0.202 |
| SSI first definition (start from second diagnoses)^b^ (number of infections per 1,000 discharges) | | | | | | | | | |  |  |  |  |  |  |  |
|  | 23.89 | 18.98 | 23.53 | 28.26 | 24.82 | 20.22 | 25.29 | 21.63 | 28.02 | 20.93 | 23.94 | 30.03 | 25.5 | 14.95 | 21.91 | 23.42 |
| SSI first definition (all diagnoses)^c^ | | | | | |  |  |  |  |  |  |  |  |  |  |  |
|  | 23.89 | 19.8 | 23.53 | 28.26 | 25.71 | 21.06 | 26.1 | 21.63 | 28.99 | 20.93 | 23.94 | 30.03 | 26.44 | 14.95 | 21.91 | 23.77 |
| SSI second definition (start from second diagnoses)^d^ | | | | | |  |  |  |  |  |  |  |  |  |  |  |
|  | 22.29 | 23.93 | 26.05 | 29.09 | 20.39 | 21.9 | 22.02 | 22.49 | 29.95 | 25.48 | 24.82 | 29.03 | 23.61 | 18.69 | 23.9 | 24.19 |
| SSI second definition (all diagnoses)^e^ | | | | | |  |  |  |  |  |  |  |  |  |  |  |
|  | 22.29 | 24.75 | 26.05 | 29.09 | 20.39 | 22.75 | 22.84 | 22.49 | 30.92 | 25.48 | 24.82 | 29.03 | 24.55 | 18.69 | 23.9 | 24.48 |
| SSI third definition (start from second diagnoses)^f^ | | | | | |  |  |  |  |  |  |  |  |  |  |  |
|  | 16.72 | 14.85 | 15.13 | 17.46 | 14.18 | 16.85 | 15.5 | 17.3 | 22.22 | 16.38 | 15.07 | 17.02 | 17 | 9.35 | 16.93 | 16.1 |
| SSI third definition (all diagnoses)^g^ | | | | | |  |  |  |  |  |  |  |  |  |  |  |
|  | 16.72 | 14.85 | 15.13 | 17.46 | 15.07 | 17.69 | 16.31 | 17.3 | 22.22 | 16.38 | 15.07 | 17.02 | 17.94 | 9.35 | 16.93 | 16.34 |
| **Colon surgery (% of discharges that underwent this procedures)** | | | | | | | | | | | | | | | | |
|  | 1.167 | 1.201 | 1.216 | 1.172 | 1.139 | 1.239 | 1.292 | 1.214 | 1.217 | 1.207 | 1.227 | 1.128 | 1.109 | 1.170 | 1.144 | 1.189 |
| SSI first definition (start from second diagnoses)^h^ | | | | | |  |  |  |  |  |  |  |  |  |  |  |
|  | 159.69 | 170.78 | 164.82 | 162.89 | 172.23 | 160.8 | 163.32 | 165.4 | 164.08 | 172.59 | 169.77 | 172.44 | 171.14 | 167.12 | 156.92 | 166.27 |
| SSI first definition (all diagnoses)^i^ | | | | | |  |  |  |  |  |  |  |  |  |  |  |
|  | 166.67 | 177.13 | 170.21 | 167.84 | 178.14 | 165.73 | 168.19 | 171.77 | 169.84 | 179.07 | 174.95 | 177.55 | 175.87 | 174.88 | 161.69 | 171.98 |
| SSI second definition (start from second diagnoses) | | | | | |  |  |  |  |  |  |  |  |  |  |  |
|  | 123.65 | 133.55 | 133.8 | 129.83 | 135.16 | 126.94 | 132.38 | 133.57 | 131.62 | 135.24 | 135.78 | 136.68 | 138.8 | 132.6 | 114.11 | 131.65 |
| SSI second definition (all diagnoses) | | | | | |  |  |  |  |  |  |  |  |  |  |  |
|  | 126.5 | 137.1 | 136.35 | 132.08 | 139.44 | 129.4 | 134.81 | 136.6 | 134.87 | 138.7 | 137.92 | 138.59 | 140.18 | 135.95 | 116.32 | 134.4 |
| SSI third definition (start from second diagnoses) | | | | | |  |  |  |  |  |  |  |  |  |  |  |
|  | 49.57 | 49.34 | 51.99 | 47.78 | 47.12 | 43.28 | 49.86 | 46.09 | 47.51 | 45.93 | 45.87 | 45.03 | 40.12 | 38.17 | 34.62 | 45.62 |
| SSI third definition (all diagnoses) | | | | | |  |  |  |  |  |  |  |  |  |  |  |
|  | 52.99 | 53.04 | 55.59 | 51.99 | 51.26 | 46.32 | 53.87 | 50.49 | 51.35 | 50.45 | 51.05 | 49.66 | 43.93 | 42.12 | 39.06 | 49.66 |
| **Cardiac implantable electronic device (% of discharges that underwent this procedures)** | | | | | | | | | | | | | | | | |
|  | 1.533 | 1.572 | 1.561 | 1.412 | 1.386 | 1.529 | 1.518 | 1.348 | 1.330 | 1.421 | 1.429 | 1.289 | 1.288 | 1.376 | 1.366 | 1.424 |
| SSI first definition (start from second diagnoses)^l^ | | | | | |  |  |  |  |  |  |  |  |  |  |  |
|  | 4.77 | 5.08 | 4.78 | 4.12 | 6.07 | 6.21 | 4.02 | 5.33 | 5.4 | 5.25 | 4.45 | 4.47 | 4.73 | 5.95 | 6.14 | 5.11 |
| SSI first definition (all diagnoses)^m^ | | | | | |  |  |  |  |  |  |  |  |  |  |  |
|  | 26.24 | 27.77 | 27.54 | 32.43 | 30.84 | 30.48 | 28.27 | 31.28 | 29.56 | 29.05 | 37.04 | 32.83 | 26.27 | 29.73 | 35.14 | 30.16 |
| SSI second definition (start from second diagnoses) | | | | | |  |  |  |  |  |  |  |  |  |  |  |
|  | 4.23 | 7 | 6.3 | 6.74 | 8.26 | 5.86 | 5.73 | 6.56 | 7.83 | 7.42 | 10.47 | 6.29 | 6.31 | 7.37 | 6.14 | 6.8 |
| SSI second definition (all diagnoses) | | | | | |  |  |  |  |  |  |  |  |  |  |  |
|  | 4.77 | 7.45 | 6.88 | 7.86 | 8.98 | 6.21 | 6.34 | 7.51 | 8.64 | 8.06 | 10.86 | 6.85 | 6.83 | 7.76 | 6.71 | 7.41 |
| SSI third definition (start from second diagnoses) | | | | | |  |  |  |  |  |  |  |  |  |  |  |
|  | 6.5 | 7.22 | 7.58 | 6.36 | 9.96 | 8.91 | 5.48 | 7.51 | 7.83 | 7.42 | 7.2 | 6.01 | 6.96 | 7.76 | 7.71 | 7.37 |
| SSI third definition (all diagnoses) | | | | | |  |  |  |  |  |  |  |  |  |  |  |
|  | 28.3 | 30.14 | 31.16 | 35.05 | 35.45 | 33.64 | 29.86 | 33.6 | 32.94 | 31.73 | 39.92 | 34.79 | 29.16 | 31.67 | 37.28 | 32.85 |
| **Laparoscopic cholecystectomy and laparoscopic appendectomy (% of discharges that underwent this procedures)** | | | | | | | | | | | | | | | | |
|  | 0.835 | 0.895 | 0.958 | 0.849 | 0.784 | 0.902 | 0.935 | 0.865 | 0.840 | 0.860 | 0.924 | 0.834 | 0.799 | 0.863 | 0.909 | 0.869 |
| SSI first definition (start from second diagnoses)^j^ | | | | | |  |  |  |  |  |  |  |  |  |  |  |
|  | 14.34 | 15.06 | 15.03 | 15.14 | 16.32 | 15.3 | 13.46 | 17.88 | 18.39 | 17.76 | 16.6 | 18.36 | 17.15 | 15.46 | 16.1 | 16.11 |
| SSI first definition (all diagnoses)^k^ | | | | | |  |  |  |  |  |  |  |  |  |  |  |
|  | 14.74 | 15.25 | 15.22 | 15.35 | 16.54 | 15.3 | 13.85 | 18.52 | 18.39 | 17.97 | 16.6 | 18.57 | 17.15 | 15.46 | 16.53 | 16.32 |
| SSI second definition (start from second diagnoses) | | | | | |  |  |  |  |  |  |  |  |  |  |  |
|  | 16.73 | 15.25 | 16.74 | 16.39 | 16.97 | 15.3 | 14.25 | 19.8 | 16.89 | 17.12 | 17.81 | 20.09 | 17.37 | 15.88 | 17.18 | 16.88 |
| SSI second definition (all diagnoses) | | | | | |  |  |  |  |  |  |  |  |  |  |  |
|  | 17.33 | 15.45 | 17.12 | 16.59 | 17.4 | 15.3 | 15.24 | 20.65 | 17.32 | 17.55 | 18.42 | 20.95 | 17.58 | 16.29 | 17.82 | 17.36 |
| SSI third definition (start from second diagnoses) | | | | | |  |  |  |  |  |  |  |  |  |  |  |
|  | 8.36 | 7.33 | 8.18 | 8.09 | 6.01 | 8.55 | 7.32 | 8.09 | 6.63 | 7.19 | 7.69 | 9.07 | 6.57 | 6.6 | 7.52 | 7.56 |
| SSI third definition (all diagnoses) | | | | | |  |  |  |  |  |  |  |  |  |  |  |
|  | 9.36 | 7.53 | 8.37 | 8.92 | 6.44 | 9.14 | 8.71 | 9.37 | 7.7 | 7.61 | 9.51 | 10.15 | 6.99 | 7.42 | 8.59 | 8.39 |
| **Orthopedic procedures (% of discharges that underwent this procedures)** | | | | | | | | | | | | | | | | |
|  | 1.018 | 1.069 | 1.105 | 1.082 | 1.078 | 1.166 | 1.229 | 1.196 | 1.215 | 1.265 | 1.339 | 1.223 | 1.215 | 1.268 | 1.364 | 1.186 |
| SSI first definition (start from second diagnoses)^l^ | | | | | |  |  |  |  |  |  |  |  |  |  |  |
|  | 2.78 | 2.16 | 3.13 | 4.07 | 2.97 | 3.07 | 3.61 | 1.54 | 2.66 | 2.3 | 2.51 | 1.47 | 1.95 | 3.65 | 2.43 | 2.68 |
| SSI first definition (all diagnoses)^m^ | | | | | |  |  |  |  |  |  |  |  |  |  |  |
|  | 3.92 | 3.98 | 4.94 | 5.86 | 4.37 | 5.84 | 5.72 | 3.23 | 4.73 | 4.46 | 4.19 | 2.5 | 3.48 | 5.47 | 4.44 | 4.47 |
| SSI second definition (start from second diagnoses) | | | | | |  |  |  |  |  |  |  |  |  |  |  |
|  | 4.73 | 3.15 | 4.78 | 4.23 | 4.37 | 4.76 | 5.12 | 2.93 | 3.25 | 3.31 | 4.47 | 3.24 | 3.34 | 4.35 | 2.72 | 3.9 |
| SSI second definition (all diagnoses) | | | | | |  |  |  |  |  |  |  |  |  |  |  |
|  | 5.22 | 3.82 | 5.44 | 5.7 | 5 | 5.69 | 6.02 | 3.23 | 3.99 | 4.74 | 5.45 | 3.68 | 4.04 | 4.91 | 3.72 | 4.7 |
| SSI third definition (start from second diagnoses) | | | | | |  |  |  |  |  |  |  |  |  |  |  |
|  | 5.39 | 3.98 | 5.93 | 5.05 | 4.68 | 3.84 | 5.72 | 2.46 | 4.58 | 3.74 | 4.75 | 2.8 | 3.06 | 5.89 | 5.15 | 4.46 |
| SSI third definition (all diagnoses) | | | | | |  |  |  |  |  |  |  |  |  |  |  |
|  | 6.37 | 5.81 | 7.91 | 7 | 6.09 | 6.76 | 7.83 | 4.62 | 6.65 | 6.18 | 6.7 | 3.98 | 4.59 | 8 | 7.01 | 6.36 |
| **All other procedure (% of discharges that underwent this procedures)** | | | | | | | | | | | | | | | | |
|  | 24.866 | 25.516 | 25.680 | 24.769 | 24.525 | 26.029 | 26.666 | 25.853 | 25.769 | 25.954 | 26.579 | 25.008 | 24.607 | 25.841 | 26.421 | 25.582 |
| SSI first definition (start from second diagnoses)^n^ | | | | | |  |  |  |  |  |  |  |  |  |  |  |
|  | 18.74 | 19.84 | 20.82 | 19.65 | 20.14 | 19.16 | 20.4 | 20.73 | 20.31 | 20.07 | 20.56 | 20.11 | 19.85 | 19.71 | 17.08 | 19.82 |
| SSI first definition (all diagnoses)^o^ | | | | | |  |  |  |  |  |  |  |  |  |  |  |
|  | 23.93 | 25.3 | 26.34 | 24.79 | 25.36 | 24.57 | 26.03 | 26.43 | 25.29 | 25.43 | 26.44 | 25.11 | 24.69 | 24.84 | 22.51 | 25.14 |
| SSI second definition (start from second diagnoses) | | | | | |  |  |  |  |  |  |  |  |  |  |  |
|  | 11.88 | 12.1 | 12.76 | 11.75 | 11.41 | 10.92 | 11.84 | 11.52 | 11.31 | 11.25 | 11.18 | 11.14 | 10.37 | 10.44 | 9.72 | 11.31 |
| SSI second definition (all diagnoses) | | | | | |  |  |  |  |  |  |  |  |  |  |  |
|  | 26.79 | 28.43 | 29.68 | 27.72 | 26.74 | 27.51 | 29.27 | 28.13 | 27.27 | 28.68 | 29.62 | 27.89 | 26.3 | 27.67 | 27.4 | 27.93 |
| ^a^Percentage of patients that had abdominal hysterectomy. ^b^SSIs are identified by ICD-9 codes: 567.22, 682.2, 998.31, 998.32, 998.51, and 998.59, starting from the second diagnoses to prevent possible present-on-admission infections. ^c^Same definitions to b, but the identification includes the first diagnoses. ^d^SSI definitions are ICD-9 codes: 998.5, 998.51, 998.59, 996.69, 567.2–567.29, 567.9, 567.3–567.39, 682.2, and 682.9. Identification starts from the second diagnoses to prevent possible present-on-admission infections. ^e^Same definitions to d, but the identification includes the first diagnoses. ^f^SSI definitions are ICD-9 codes: 998.5, 998.51, 998.59, 996.6-996.69. Identification starts from the second diagnoses to prevent possible present-on-admission infections. ^g^Same definitions to f, but the identification includes the first diagnoses. | | | | | | | | | | | | | | | | |
| ^h^SSI for colon surgeries 567.21, 567.22, 567.29, 567.38, 569.5, 596.61, 596.81, 682.2, 879.9, 998.31, 998.32, 998.51, 998.59, 998.6, 54.0, 54.11, 54.19, 86.04, 86.22, and 86.28. Identification starts from the second diagnoses to prevent possible present-on-admission infections. ^i^Same definitions to h, but the identification includes the first diagnoses. | | | | | | | | | | | | | | | | |
| ^j^Laparoscopic cholecystectomy and laparoscopic appendectomy SSI: 567, 567.2, 567.21, 567.22, 567.23, 567.29, 567.3, 567.38, 567.39, 567.8, 567.81, 567.89, 567.9, 682.2 Identification starts from the second diagnoses to prevent possible present-on-admission infections. ^k^Same definitions to j, but the identification includes the first diagnoses. | | | | | | | | | | | | | | | | |
| ^l^Orthopedic procedures, cardiac implantable electronic device SSI: 996.61, 998.59 Identification starts from the second diagnoses to prevent possible present-on-admission infections. ^m^Same definitions to l, but the identification includes the first diagnoses. | | | | | | | | | | | | | | | | |
| ^n^SSI for procedures other than abdominal hysterectomy and colon: 998.5, 998.51, 998.59, 996.6-996.69 Identification starts from the second diagnoses to prevent possible present-on-admission infections. ^o^Same definitions to n, but the identification includes the first diagnoses. | | | | | | | | | | | | | | | | |
