# Supplementary material for: Evaluation of hospital-acquired conditions reduction program in surgical procedures
Source: PLoS One. 2025 Nov 21;20(11):e0337072. doi: 10.1371/journal.pone.0337072 (PMC12637954; doi:10.1371/journal.pone.0337072)
Supplement: S4 Table — Reported coefficients are changes per 1,000 discharges. (DOCX) [file pone.0337072.s005.docx]

S4 Table lists the DID estimates from Equation one in the “DID Model and Statistical Analysis” section when the alternative procedure and SSI identifications are used. Reported coefficients are changes per 1,000 discharges. The cut-off time in the DID estimate is October 2014, when the financial penalties began. The table only reported the DID estimates, i.e., the interaction term between the post-policy dummy variable and the dummy variable for the treatment procedure. For columns one and two, the treatment dummy variable is the indicator for abdominal hysterectomy. Columns three and four are for colon surgeries.

The two treatment procedures, abdominal hysterectomy and colon surgeries, are each compared with the control procedures: cardiac implantable electronic device, laparoscopic cholecystectomy and appendectomy, orthopedic procedures, and all other procedures. The two procedures in each comparison are defined either by the primary (i.e., the first) ICD-9 procedure codes or by all procedures listed in each discharge. The SSIs are identified either by all codes listed for each observation or by all codes excluding the primary diagnosis codes. Detailed ICD codes are listed in S3 Table footnote, “Treatment and Control Outcome Variables” and “Sensitivity Analysis” sections in the manuscript.

Most of the estimates in the S4 Table are insignificant. The only exceptions are for colon surgeries when SSIs are defined by the third definition: 998.5, 998.51, 998.59, 996.6-996.69. This definition is more general than procedure-specific, as in definitions one and two. Overall, the results suggest no direct impact from HACRP on SSIs in abdominal hysterectomy and colon surgeries. The other two cut-off times, January 2013 and August 2013, have similar results (not reported here. Available upon request).

**S4 Table. DID estimates by comparing treatment procedures to control procedures.**

|  | (1) | (2) | (3) | (4) |
| --- | --- | --- | --- | --- |
|  | **Abdominal hysterectomy vs laparoscopic cholecystectomy and appendectomy (primary)** | **Abdominal hysterectomy vs laparoscopic cholecystectomy and appendectomy (all proc)** | **Colon vs laparoscopic cholecystectomy and appendectomy (primary)** | **Colon vs laparoscopic cholecystectomy and appendectomy (all)** |
|  | SSI definition 1, all diag | SSI definition 1, all diag | SSI definition 1, all diag | SSI definition 1, all diag |
| Post*treatment | -1.322 | -1.505 | 3.323 | 1.143 |
|  | (3.018) | (3.012) | (3.300) | (3.192) |
|  | [ -7.237, 4.593] | [ -7.409, 4.399] | [ -3.145, 9.791] | [ -5.113, 7.399] |
|  | 71358 | 83809 | 134980 | 159844 |
|  |  |  |  |  |
|  | SSI definition 2, all diag | SSI definition 2, all diag | SSI definition 2, all diag | SSI definition 2, all diag |
| Post*treatment | -2.665 | -1.877 | 1.509 | -1.345 |
|  | (2.952) | (3.079) | (2.980) | (2.867) |
|  | [ -8.451, 3.121] | [ -7.912, 4.158] | [ -4.332, 7.35] | [ -6.964, 4.274] |
|  | 71358 | 83809 | 134980 | 159844 |
|  |  |  |  |  |
|  | SSI definition 3, all diag | SSI definition 3, all diag | SSI definition 3, all diag | SSI definition 3, all diag |
| Post*treatment | -1.385 | -1.465 | -6.223*** | -7.650*** |
|  | (2.479) | (2.399) | (1.765) | (1.736) |
|  | [ -6.244, 3.474] | [ -6.167, 3.237] | [ -9.682, -2.764] | [ -11.053, -4.247] |
|  | 71358 | 83809 | 134980 | 159844 |
|  |  |  |  |  |
|  | Definition 1 from 2nd diag | Definition 1 from 2nd diag | Definition 1 from 2nd diag | Definition 1 from 2nd diag |
| Post*treatment | -1.717 | -1.464 | 3.352 | 1.280 |
|  | (2.996) | (2.990) | (3.276) | (3.135) |
|  | [ -7.589, 4.155] | [ -7.324, 4.396] | [ -3.069, 9.773] | [ -4.865, 7.425] |
|  | 71358 | 83809 | 134980 | 159844 |
|  |  |  |  |  |
|  | Definition 2 from 2nd diag | Definition 2 from 2nd diag | Definition 2 from 2nd diag | Definition 2 from 2nd diag |
| Post*treatment | -3.180 | -1.857 | 1.544 | -0.738 |
|  | (2.929) | (3.059) | (2.955) | (2.830) |
|  | [ -8.921, 2.561] | [ -7.853, 4.139] | [ -4.248, 7.336] | [ -6.285, 4.809] |
|  | 71358 | 83809 | 134980 | 159844 |
|  |  |  |  |  |
|  | Definition 3 from 2nd diag | Definition 3 from 2nd diag | Definition 3 from 2nd diag | Definition 3 from 2nd diag |
| Post*treatment | -1.337 | -1.491 | -6.614*** | -7.660*** |
|  | (2.471) | (2.374) | (1.715) | (1.675) |
|  | [ -6.18, 3.506] | [ -6.144, 3.162] | [ -9.975, -3.253] | [ -10.943, -4.377] |
|  | 71358 | 83809 | 134980 | 159844 |
|  | **Abdominal hysterectomy vs orthopedic procedures (primary procedure)** | **Abdominal hysterectomy vs orthopedic procedures (all procedure)** | **Colon surgeries vs orthopedic procedures (primary procedure)** | **Colon surgeries vs orthopedic procedures (all procedure)** |
|  | SSI definition 1, all diag | SSI definition 1, all diag | SSI definition 1, all diag | SSI definition 1, all diag |
| Post*treatment | 0.698 | -0.100 | 4.247 | 1.716 |
|  | (2.821) | (2.808) | (3.160) | (3.038) |
|  | [ -4.831, 6.227] | [ -5.604, 5.404] | [ -1.947, 10.441] | [ -4.238, 7.67] |
|  | 94797 | 108231 | 158419 | 185473 |
|  |  |  |  |  |
|  | SSI definition 2, all diag | SSI definition 2, all diag | SSI definition 2, all diag | SSI definition 2, all diag |
| Post*treatment | -0.404 | -0.126 | 2.522 | -0.565 |
|  | (2.767) | (2.866) | (2.815) | (2.693) |
|  | [ -5.827, 5.019] | [ -5.743, 5.491] | [ -2.995, 8.039] | [ -5.843, 4.713] |
|  | 94797 | 108231 | 158419 | 185473 |
|  |  |  |  |  |
|  | SSI definition 3, all diag | SSI definition 3, all diag | SSI definition 3, all diag | SSI definition 3, all diag |
| Post*treatment | -0.352 | -1.208 | -5.447*** | -7.445*** |
|  | (2.409) | (2.342) | (1.680) | (1.655) |
|  | [ -5.074, 4.37] | [ -5.798, 3.382] | [ -8.74, -2.154] | [ -10.689, -4.201] |
|  | 94797 | 108231 | 158419 | 185473 |
|  |  |  |  |  |
|  | Definition 1 from 2nd diag | Definition 1 from 2nd diag | Definition 1 from 2nd diag | Definition 1 from 2nd diag |
| Post*treatment | 0.153 | -0.189 | 4.170 | 1.763 |
|  | (2.783) | (2.769) | (3.123) | (2.975) |
|  | [ -5.302, 5.608] | [ -5.616, 5.238] | [ -1.951, 10.291] | [ -4.068, 7.594] |
|  | 94797 | 108231 | 158419 | 185473 |
|  |  |  |  |  |
|  | Definition 2 from 2nd diag | Definition 2 from 2nd diag | Definition 2 from 2nd diag | Definition 2 from 2nd diag |
| Post*treatment | -0.900 | -0.266 | 2.638 | -0.078 |
|  | (2.735) | (2.838) | (2.792) | (2.664) |
|  | [ -6.261, 4.461] | [ -5.828, 5.296] | [ -2.834, 8.11] | [ -5.299, 5.143] |
|  | 94797 | 108231 | 158419 | 185473 |
|  |  |  |  |  |
|  | Definition 3 from 2nd diag | Definition 3 from 2nd diag | Definition 3 from 2nd diag | Definition 3 from 2nd diag |
| Post*treatment | -0.569 | -1.462 | -6.072*** | -7.670*** |
|  | (2.392) | (2.300) | (1.620) | (1.582) |
|  | [ -5.257, 4.119] | [ -5.97, 3.046] | [ -9.247, -2.897] | [ -10.771, -4.569] |
|  | 94797 | 108231 | 158419 | 185473 |
|  | **Abdominal hysterectomy vs Cardiac implantable electronic device (primary procedure)^a^** | **Abdominal hysterectomy vs Cardiac implantable electronic device (all proc)^c^** | **Colon surgeries vs. Cardiac implantable electronic device (primary proc)** | **Colon surgeries vs Cardiac implantable electronic device (all procedure)** |
|  | SSI definition 1, all diag^b^ | SSI definition 1, all diag | SSI definition 1, all diag^d^ | SSI definition 1, all diag |
| Post*treatment | 3.132 | -1.241 | -2.884 | 2.381 |
|  | (3.473) | (3.085) | (2.607) | (3.351) |
|  | [ -3.675, 9.939] | [ -7.288, 4.806] | [ -7.994, 2.226] | [ -4.187, 8.949] |
|  | 76797 | 126726 | 140419 | 203842 |
|  |  |  |  |  |
|  | SSI definition 2, all diag^e^ | SSI definition 2, all diag | SSI definition 2, all diag | SSI definition 2, all diag |
| Post*treatment | -0.461 | -0.083 | 3.967 | 1.542 |
|  | (2.845) | (2.894) | (2.911) | (2.743) |
|  | [ -6.037, 5.115] | [ -5.755, 5.589] | [ -1.739, 9.673] | [ -3.834, 6.918] |
|  | 76797 | 126726 | 140419 | 203842 |
|  |  |  |  |  |
|  | SSI definition 3, all diag | SSI definition 3, all diag | SSI definition 3, all diag | SSI definition 3, all diag |
| Post*treatment | 2.475 | -1.698 | 3.568 | -7.428*** |
|  | (3.131) | (2.636) | (3.795) | (2.106) |
|  | [ -3.662, 8.612] | [ -6.865, 3.469] | [ -3.87, 11.006] | [ -11.556, -3.3] |
|  | 76797 | 126726 | 140419 | 203842 |
|  |  |  |  |  |
|  | Definition 1 from 2nd diag | Definition 1 from 2nd diag | Definition 1 from 2nd diag | Definition 1 from 2nd diag |
| Post*treatment | -0.321 | -0.750 | 4.692 | 2.843 |
|  | (2.827) | (2.790) | (3.182) | (3.003) |
|  | [ -5.862, 5.22] | [ -6.218, 4.718] | [ -1.545, 10.929] | [ -3.043, 8.729] |
|  | 76797 | 126726 | 140419 | 203842 |
|  |  |  |  |  |
|  | Definition 2 from 2nd diag | Definition 2 from 2nd diag | Definition 2 from 2nd diag | Definition 2 from 2nd diag |
| Post*treatment | -0.903 | -0.228 | 4.079 | 1.995 |
|  | (2.829) | (2.880) | (2.888) | (2.710) |
|  | [ -6.448, 4.642] | [ -5.873, 5.417] | [ -1.581, 9.739] | [ -3.317, 7.307] |
|  | 76797 | 126726 | 140419 | 203842 |
|  |  |  |  |  |
|  | Definition 3 from 2nd diag | Definition 3 from 2nd diag | Definition 3 from 2nd diag | Definition 3 from 2nd diag |
|  | -0.460 | -1.340 | -6.026*** | -7.206*** |
|  | (2.474) | (2.330) | (1.725) | (1.620) |
|  | [ -5.309, 4.389] | [ -5.907, 3.227] | [ -9.407, -2.645] | [ -10.381, -4.031] |
|  | 76797 | 126726 | 140419 | 203842 |
|  | **Abdominal hysterectomy vs other procedures (all procedures)** | | **Colon surgeries vs other procedures (all procedures)** | |
|  | SSI definition 1, all diag |  | SSI definition 1, all diag |  |
| Post*treatment | 1.856 |  | 3.986 |  |
|  | (3.031) |  | (3.194) |  |
|  | [ -4.085, 7.797] |  | [ -2.274, 10.246] |  |
|  | 1988204 |  | 1988338 |  |
|  |  |  |  |  |
|  | SSI definition 2, all diag |  | SSI definition 2, all diag |  |
| Post*treatment | 0.426 |  | 0.133 |  |
|  | (2.894) |  | (2.721) |  |
|  | [ -5.246, 6.098] |  | [ -5.2, 5.466] |  |
|  | 1988204 |  | 1988338 |  |
|  |  |  |  |  |
|  | Definition 1 from 2nd diag |  | Definition 1 from 2nd diag |  |
| Post*treatment | 1.536 |  | 4.241 |  |
|  | (3.018) |  | (3.149) |  |
|  | [ -4.379, 7.451] |  | [ -1.931, 10.413] |  |
|  | 1988204 |  | 1988338 |  |
|  |  |  |  |  |
|  | Definition 2 from 2nd diag |  | Definition 2 from 2nd diag |  |
| Post*treatment | 0.448 |  | 0.735 |  |
|  | (2.865) |  | (2.688) |  |
|  | [ -5.167, 6.063] |  | [ -4.533, 6.003] |  |
|  | 1988204 |  | 1988338 |  |
| ^a^procedure defined by primary (i.e., first) ICD-9 procedure code. ^b^ SSIs for Abdominal hysterectomy are identified by ICD-9 codes: 567.22, 682.2, 998.31, 998.32, 998.51, and 998.59. SSIs for Cardiac implantable electronic device are 996.61, 998.59. Identification examines all ICD-9 codes, including the first, i.e., primary, codes. ^c^procedure defined by all ICD-9 procedure codes. | | | | |
| ^d^ SSI for colon surgeries 567.21, 567.22, 567.29, 567.38, 569.5, 596.61, 596.81, 682.2, 879.9, 998.31, 998.32, 998.51, 998.59, 998.6, 54.0, 54.11, 54.19, 86.04, 86.22, and 86.28. Identification examines all ICD-9 codes, including the first, i.e., primary, codes. | | | | |
| ^e^SSI definitions for Abdominal hysterectomy are ICD-9 codes: 998.5, 998.51, 998.59, 996.69, 567.2–567.29, 567.9, 567.3–567.39, 682.2, and 682.9 For other definitions, please refer to S3 Table footnote, and “Treatment and Control Outcome Variables” and “Sensitivity Analysis” sections. | | | | |
