# Supplementary material for: Evaluation of hospital-acquired conditions reduction program in surgical procedures
Source: PLoS One. 2025 Nov 21;20(11):e0337072. doi: 10.1371/journal.pone.0337072 (PMC12637954; doi:10.1371/journal.pone.0337072)
Supplement: S5 Table — (DOCX) [file pone.0337072.s006.docx]

**S5 Table DID estimates by comparing treatment procedures with control procedures (sample period 2012-2017)**

|  | (1) | (2) | (3) | (4) |
| --- | --- | --- | --- | --- |
| **Abdominal hysterectomy vs.** | **lap chole and lap appy^a^** | **orthopedic procedure** | **CIED^b^** | **other procedures** |
| Post*treatment | 1.203^c^ | 0.701 | 1.981 | 2.469 |
|  | (4.776)^d^ | (4.459) | (5.525) | (4.701) |
|  | [-8.158, 10.564]^e^ | [-8.039, 9.441] | [-8.849, 12.811] | [-6.745, 11.684] |
|  | 114741f | 159511 | 132824 | 3270507 |
|  |  |  |  |  |
| **Colon surgeries vs.** | **lap chole and lap appy^a^** | **orthopedic procedure** | **CIED^b^** | **other procedures** |
| Post*treatment | 6.514 | 5.866 | 6.227 | 1.926 |
|  | (5.121) | (4.849) | (5.830) | (4.735) |
|  | [-3.524, 16.552] | [-3.638, 15.370] | [-5.200, 17.654] | [-7.354, 11.206] |
|  | 224940 | 269710 | 243023 | 3281526 |
| ^a^Laparoscopic cholecystectomy and laparoscopic appendectomy  ^b^Cardiac Implantable Electronic Device  ^c^ coefficient estimate ^d^ standard error ^e^95% confidence intervals ^f^number of observations  For definitions on procedures and SSIs, please refer to Table 2 footnote, and the section Treatment and Control Outcome Variables. All models control patient and hospital characteristics and time trend. | | | | |
