# Supplementary material for: Evaluation of hospital-acquired conditions reduction program in surgical procedures
Source: PLoS One. 2025 Nov 21;20(11):e0337072. doi: 10.1371/journal.pone.0337072 (PMC12637954; doi:10.1371/journal.pone.0337072)
Supplement: S7 Table — (DOCX) [file pone.0337072.s008.docx]

**Supporting information**

**S7 Table interrupted time series for abdominal hysterectomy and colon surgeries (sample period 2012-2015)**

|  | (1) | (2) | (3) | (4) | (5) | (6) |
| --- | --- | --- | --- | --- | --- | --- |
| **Abdominal hysterectomy (primary) ^a^** | SSI definition 1, from all diag ^e^ | SSI definition 2, from all diag ^f^ | SSI definition 3, from all diag^g^ | SSI Definition 1 from 2nd diag^h^ | SSI Definition 2 from 2nd diag^i^ | SSI Definition 3 from 2nd diag^j^ |
| *Post* |  |  |  |  |  |  |
| Coeff. | 2.696 | -0.174 | -2.727 | 2.159 | -0.779 | -2.660 |
| SE | (5.265) | (5.220) | (4.537) | (5.240) | (5.208) | (4.523) |
| CI | [-7.623, 13.016] | [-10.407, 10.058] | [-11.621, 6.166] | [-8.111, 12.429] | [-10.987, 9.429] | [-11.525, 6.205] |
| *Post*Trend* |  |  |  |  |  |  |
| Coeff. | -1.491 | -0.854 | 1.003 | -1.363 | -0.708 | 0.985 |
| SE | (2.276) | (2.257) | (1.962) | (2.266) | (2.252) | (1.956) |
| CI | [-5.953, 2.971] | [-5.279, 3.570] | [-2.843, 4.848] | [-5.804, 3.078] | [-5.122, 3.706] | [-2.849, 4.818] |
| N of obs. | 11364 | 11364 | 11364 | 11364 | 11364 | 11364 |
| **Abdominal hysterectomy (all proc) ^b^** | SSI definition 1, from all diag | SSI definition 2, from all diag | SSI definition 3, from all diag | SSI Definition 1 from 2nd diag | SSI Definition 2 from 2nd diag | SSI Definition 3 from 2nd diag |
| *Post* |  |  |  |  |  |  |
| Coeff. | 2.511 | -0.0551 | -3.346 | 2.596 | -0.00981 | -3.529 |
| SE | (5.074) | (5.158) | (4.292) | (5.042) | (5.133) | (4.262) |
| CI | [-7.434, 12.456] | [-10.165, 10.055] | [-11.759, 5.066] | [-7.286, 12.479] | [-10.071, 10.051] | [-11.883, 4.824] |
| *Post*Trend* |  |  |  |  |  |  |
| Coeff. | -1.890 | -0.610 | 0.585 | -1.764 | -0.470 | 0.678 |
| SE | (2.206) | (2.243) | (1.866) | (2.193) | (2.232) | (1.853) |
| CI | [-6.215, 2.434] | [-5.006, 3.787] | [-3.074, 4.243] | [-6.062, 2.534] | [-4.845, 3.905] | [-2.955, 4.311] |
| N of obs. | 15722 | 15722 | 15722 | 15722 | 15722 | 15722 |
| **Colon surgeries (primary)^c^** | SSI definition 1, all diag^k^ | SSI definition 2, all diag ^l^ | SSI definition 3, all diag ^m^ | SSI Definition 1 from 2nd diag | SSI Definition 2 from 2nd diag | SSI Definition 3 from 2nd diag |
| *Post* |  |  |  |  |  |  |
| Coeff. | 5.918 | 2.920 | -1.563 | 6.069 | 3.506 | -2.103 |
| SE | (5.450) | (4.914) | (3.022) | (5.402) | (4.875) | (2.926) |
| CI | [-4.764, 16.599] | [-6.711, 12.551] | [-7.486, 4.359] | [-4.519, 16.657] | [-6.050, 13.062] | [-7.838, 3.632] |
| *Post*Trend* |  |  |  |  |  |  |
| Coeff. | -0.241 | -2.956 | -0.429 | -0.729 | -3.467* | -0.123 |
| SE | (2.302) | (2.076) | (1.277) | (2.282) | (2.060) | (1.236) |
| CI | [-4.754, 4.272] | [-7.025, 1.113] | [-2.931, 2.073] | [-5.202, 3.744] | [-7.504, 0.570] | [-2.546, 2.300] |
| N of obs. | 75375 | 75375 | 75375 | 75375 | 75375 | 75375 |
| **Colon surgeries**  **(all proc)^d^** | SSI definition 1, all diag | SSI definition 2, all diag | SSI definition 3, all diag | SSI Definition 1 from 2nd diag | SSI Definition 2 from 2nd diag | SSI Definition 3 from 2nd diag |
| *Post* |  |  |  |  |  |  |
| Coeff. | 0.224 | -0.397 | -3.477 | 0.546 | 1.057 | -2.965 |
| SE | (5.028) | (4.523) | (2.920) | (4.961) | (4.485) | (2.808) |
| CI | [-9.630, 10.077] | [-9.262, 8.468] | [-9.200, 2.246] | [-9.178, 10.270] | [-7.733, 9.847] | [-8.468, 2.538] |
| *Post*Trend* |  |  |  |  |  |  |
| Coeff. | 0.141 | -1.970 | -0.475 | -0.195 | -2.619 | -0.329 |
| SE | (2.133) | (1.919) | (1.239) | (2.105) | (1.903) | (1.191) |
| CI | [-4.039, 4.322] | [-5.731, 1.791] | [-2.903, 1.953] | [-4.320, 3.930] | [-6.348, 1.110] | [-2.663, 2.006] |
| N of obs. | 93522 | 93522 | 93522 | 93522 | 93522 | 93522 |
| Coeff. = coefficient estimates; SE = standard error; CI = 95% confidence interval  All models control for patient and hospital characteristics and time trend. | | | | | | |
| ^a^ Abdominal hysterectomy procedure defined by primary (i.e., first) ICD-9 procedure code: 68.31, 68.39, 68.41, 68.49, 68.61, and 68.69.  ^b^ Abdominal hysterectomy defined by all ICD-9 procedure codes (i.e., first and the rest of procedures).  ^c^ Colon surgeries if their first, i.e., primary, ICD-9 procedure codes were 17.31-17.36, 17.39, 45.03, 45.26, 45.41, 45.49, 45.52, 45.71-45.76, 45.79, 45.81-45.83, 45.92-45.95, 46.03, 46.04, 46.10, 46.11, 46.13, 46.14, 46.43, 46.52, 46.75, 46.76, and 46.94.  ^d^ Colon surgeries defined by all ICD-9 procedure codes (i.e., first and the rest of codes).  ^e^ SSIs for abdominal hysterectomy, ICD-9 codes: 567.22, 682.2, 998.31, 998.32, 998.51, and 998.59, including the first diagnoses.  ^f^ Second definition for SSI, ICD-9 codes: 998.5, 998.51, 998.59, 996.69, 567.2–567.29, 567.9, 567.3–567.39, 682.2, 682.9  ^g^ Third definition for SSI, ICD-9 codes: 998.5, 998.51, 998.59, 996.6-996.69  ^h,i,j^ SSI defined similarly to e, f, g, but it excludes the first diagnoses (i.e., based on the second diagnoses and afterward).  ^K^ SSI for colon surgeries: 567.21, 567.22, 567.29, 567.38, 569.5, 596.61, 596.81, 682.2, 879.9, 998.31, 998.32, 998.51, 998.59, 998.6, 54.0, 54.11, 54.19, 86.04, 86.22, and 86.28, including the first diagnoses.  ^l, m^ second and third SSI definition, same to ^f^ and ^g^. | | | | | | |
