# Supplementary material for: Evaluation of hospital-acquired conditions reduction program in surgical procedures
Source: PLoS One. 2025 Nov 21;20(11):e0337072. doi: 10.1371/journal.pone.0337072 (PMC12637954; doi:10.1371/journal.pone.0337072)
Supplement: S8 Table — (DOCX) [file pone.0337072.s009.docx]

**Supporting information**

**S8 Table interrupted time series for abdominal hysterectomy and colon surgeries (sample period 2012-2017)**

|  | (1) | (2) | (3) | (4) | (5) | (6) |
| --- | --- | --- | --- | --- | --- | --- |
| **Abdominal hysterectomy (primary) ^a^** | SSI definition 1, from all diag ^e^ | SSI definition 2, from all diag ^f^ | SSI definition 3, from all diag^g^ | SSI Definition 1 from 2nd diag^h^ | SSI Definition 2 from 2nd diag^i^ | SSI Definition 3 from 2nd diag^j^ |
| *Post* |  |  |  |  |  |  |
| Coeff. | 2.734 | -0.200 | -2.756 | 2.189 | -0.812 | -2.688 |
| SE | (5.140) | (5.151) | (4.394) | (5.107) | (5.126) | (4.345) |
| CI | [-7.341, 12.809] | [-10.296, 9.897] | [-11.370, 5.857] | [-7.820, 12.198] | [-10.859, 9.236] | [-11.204, 5.829] |
| *Post*Trend* |  |  |  |  |  |  |
| Coeff. | -1.680 | -1.065 | 0.880 | -1.545 | -0.916 | 0.858 |
| SE | (2.221) | (2.226) | (1.899) | (2.206) | (2.215) | (1.877) |
| CI | [-6.033, 2.673] | [-5.428, 3.297] | [-2.841, 4.602] | [-5.870, 2.780] | [-5.257, 3.426] | [-2.821, 4.538] |
| N of obs. | 17346 | 17346 | 17346 | 17346 | 17346 | 17346 |
| **Abdominal hysterectomy (all proc) ^b^** | SSI definition 1, from all diag | SSI definition 2, from all diag | SSI definition 3, from all diag | SSI Definition 1 from 2nd diag | SSI Definition 2 from 2nd diag | SSI Definition 3 from 2nd diag |
| *Post* |  |  |  |  |  |  |
| Coeff. | 2.591 | -0.0657 | -3.319 | 2.670 | -0.0430 | -3.522 |
| SE | (5.015) | (5.095) | (4.152) | (4.976) | (5.053) | (4.105) |
| CI | [-7.238, 12.420] | [-10.052, 9.920] | [-11.457, 4.818] | [-7.084, 12.424] | [-9.947, 9.861] | [-11.568, 4.524] |
| *Post*Trend* |  |  |  |  |  |  |
| Coeff. | -2.005 | -0.677 | 0.540 | -1.870 | -0.539 | 0.636 |
| SE | (2.180) | (2.215) | (1.805) | (2.163) | (2.197) | (1.785) |
| CI | [-6.278, 2.268] | [-5.019, 3.665] | [-2.998, 4.078] | [-6.111, 2.370] | [-4.845, 3.767] | [-2.862, 4.134] |
| N of obs. | 23963 | 23963 | 23963 | 23963 | 23963 | 23963 |
| **Colon surgeries (primary)^c^** | SSI definition 1, all diag^k^ | SSI definition 2, all diag ^l^ | SSI definition 3, all diag ^m^ | SSI Definition 1 from 2nd diag | SSI Definition 2 from 2nd diag | SSI Definition 3 from 2nd diag |
| *Post* |  |  |  |  |  |  |
| Coeff. | 5.780 | 2.958 | -1.561 | 5.998 | 3.543 | -2.105 |
| SE | (5.596) | (4.515) | (2.781) | (5.145) | (4.476) | (2.675) |
| CI | [-5.188, 16.747] | [-5.892, 11.807] | [-7.011, 3.889] | [-4.086, 16.082] | [-5.229, 12.315] | [-7.347, 3.137] |
| *Post*Trend* |  |  |  |  |  |  |
| Coeff. | -0.240 | -2.923 | -0.400 | -0.861 | -3.432* | -0.0859 |
| SE | (2.363) | (1.907) | (1.174) | (2.173) | (1.890) | (1.130) |
| CI | [-4.872, 4.393] | [-6.661, 0.815] | [-2.702, 1.902] | [-5.121, 3.398] | [-7.137, 0.273] | [-2.300, 2.128] |
| N of obs. | 127545 | 127545 | 127545 | 127545 | 127545 | 127545 |
| **Colon surgeries**  **(all proc)^d^** | SSI definition 1, all diag | SSI definition 2, all diag | SSI definition 3, all diag | SSI Definition 1 from 2nd diag | SSI Definition 2 from 2nd diag | SSI Definition 3 from 2nd diag |
| *Post* |  |  |  |  |  |  |
| Coeff. | 0.132 | -0.411 | -3.520 | 0.556 | 1.041 | -3.001 |
| SE | (5.066) | (4.138) | (2.658) | (4.698) | (4.095) | (2.527) |
| CI | [-9.797, 10.060] | [-8.521, 7.699] | [-8.730, 1.690] | [-8.652, 9.764] | [-6.984, 9.067] | [-7.954, 1.953] |
| *Post*Trend* |  |  |  |  |  |  |
| Coeff. | 0.0919 | -1.962 | -0.495 | -0.268 | -2.611 | -0.343 |
| SE | (2.149) | (1.755) | (1.127) | (1.993) | (1.737) | (1.072) |
| CI | [-4.119, 4.303] | [-5.401, 1.478] | [-2.704, 1.715] | [-4.174, 3.638] | [-6.015, 0.793] | [-2.444, 1.758] |
| N of obs. | 166051 | 166051 | 166051 | 166051 | 166051 | 166051 |
| Coeff. = coefficient estimates; SE = standard error; CI = 95% confidence interval  All models control for patient and hospital characteristics and time trend. | | | | | | |
| ^a^ Abdominal hysterectomy procedure defined by primary (i.e., first) ICD-9 procedure code: 68.31, 68.39, 68.41, 68.49, 68.61, and 68.69.  ^b^ Abdominal hysterectomy defined by all ICD-9 procedure codes (i.e., first and the rest of procedures).  ^c^ Colon surgeries if their first, i.e., primary, ICD-9 procedure codes were 17.31-17.36, 17.39, 45.03, 45.26, 45.41, 45.49, 45.52, 45.71-45.76, 45.79, 45.81-45.83, 45.92-45.95, 46.03, 46.04, 46.10, 46.11, 46.13, 46.14, 46.43, 46.52, 46.75, 46.76, and 46.94.  ^d^ Colon surgeries defined by all ICD-9 procedure codes (i.e., first and the rest of codes).  ^e^ SSIs for abdominal hysterectomy, ICD-9 codes: 567.22, 682.2, 998.31, 998.32, 998.51, and 998.59, including the first diagnoses.  ^f^ Second definition for SSI, ICD-9 codes: 998.5, 998.51, 998.59, 996.69, 567.2–567.29, 567.9, 567.3–567.39, 682.2, 682.9  ^g^ Third definition for SSI, ICD-9 codes: 998.5, 998.51, 998.59, 996.6-996.69  ^h,i,j^ SSI defined similarly to e, f, g, but it excludes the first diagnoses (i.e., based on the second diagnoses and afterward).  ^K^ SSI for colon surgeries: 567.21, 567.22, 567.29, 567.38, 569.5, 596.61, 596.81, 682.2, 879.9, 998.31, 998.32, 998.51, 998.59, 998.6, 54.0, 54.11, 54.19, 86.04, 86.22, and 86.28, including the first diagnoses.  ^l, m^ second and third SSI definition, same to ^f^ and ^g^. | | | | | | |
